# Scoping review: mapping clinical guidelines and policy documents that address the needs of women who are dependent on drugs during the perinatal period

**DOI:** 10.1186/s12884-023-06172-6

**Published:** 2024-01-25

**Authors:** Lynne Gilmour, Louise Honeybul, Shirley Lewis, Emma Smith, Helen Cheyne, Narendra Aladangady, Brid Featherstone, Margaret Maxwell, Joanne Neale, Polly Radcliffe

**Affiliations:** 1 Nursing, Midwifery and Allied Health Professions Research Unit, Pathfoot Building, University of Stirling, Stirling, FK9 4LA Scotland; 2 Department of Behavioural and Social Sciences, University of Huddersfield, Queensgate, Huddersfield, HD1 3DH UK; 3 National Addiction Centre, Kings College London, Denmark Hill, London, SE5 8BB UK; 4 Homerton University Hospital, Homerton Row, London, E9 6SR UK

**Keywords:** Scoping review, Policy, Guidance, Pregnancy, Perinatal, Addiction, Opioids, Drugs

## Abstract

**Background:**

Women who use or are in treatment for drug use during the perinatal period often have complex needs and presenting comorbidity. Women who use opioids during pregnancy, and their infants, experience poor outcomes. Drug use by women during pregnancy is a public health priority.

This scoping review aimed to (1) map clinical guidelines, treatment protocols and good practice guidance across the UK for women who use or are in treatment for drug use during the perinatal period, (2) identify recommended best practice across health and social care for optimising outcomes and reducing inequalities for these women and (3) identify potential gaps within guidance.

**Methods:**

We followed the Joanna Briggs International (JBI) guidance on scoping reviews and PRISMA Scr extension. A registered protocol, containing a clear search strategy, inclusion, and exclusion criteria was adhered to. Reviewers double screened 25%, discussing disagreements. Data were extracted using a predefined template and charted in tables. Recommendations for best practice were organised around agreed categories.

**Results:**

Of 968 documents screened, 111 met the inclusion criteria. The documents included UK-wide, national, regional, and organisational policy documents. They varied in the degree they were relevant to women who use or are in treatment for drug use during the perinatal period, the settings to which they applied, and their intended users. Most were created without patient or public involvement and lacked any clear evidence base.

Overall, documents recommended an integrated model of care with a lead professional, clear referral pathways and information sharing between agencies. Guidance suggested referrals should be made to specialist midwives, drug, and social care services. A holistic assessment, inclusive of fathers / partners was suggested. Recent documents advocated a trauma-informed care approach. Opioid substitution therapy (OST) was recommended throughout pregnancy where required. Potential gaps were identified around provision of support for women postnatally, especially when their baby is removed from their care.

**Conclusions:**

This synthesis of recommended practice provides key information for practitioners, service providers and policy makers. It also highlights the need for guidelines to be evidence-based, informed by the experiences of women who use or are in treatment for drug use during the perinatal period, and to address the support needs of postnatal women who have their babies removed from their care.

**Supplementary Information:**

The online version contains supplementary material available at 10.1186/s12884-023-06172-6.

## Key takeaways

- This review provides a map of the guidelines and policies in this area and will be useful to practitioners seeking to navigate the broad range of available documents and identify what the best practice recommendations are
- Most documents recommended an integrated model of care with a lead professional and with clear referral pathways and information sharing protocol. More recent documents recommended working in a trauma-informed way, with practitioners recognising women’s complex histories.
- There was a range of methods used to create guidance documents and evidence drawn on to support recommendations. Guidelines should be evidence-based and written in consultation with relevant stakeholders including service users and people with lived experience.
- The review identified a gap in recommendations for the care of women who have their babies removed. More recommendations are needed for the support of this particularly vulnerable population.
- This scoping review identified the need for a systematic review assessing the effectiveness of interventions for this population.

## Background

Women who use or are in treatment for drug use during the perinatal period (pregnancy and the first year after birth) often have complex needs and co-occurring health issues including histories of trauma, such as childhood abuse, domestic abuse, mental health problems, or physical health conditions and potential elevated risk of death by suicide or drug related overdose [1, 2]. Higher numbers of women who use or are in treatment for drug use in the perinatal period live in areas of multiple deprivation and often experience low income, poor housing, and a range of health and social inequalities [3, 4]. Since 2007, across the United Kingdom (UK), there has been an increase in the number of infants becoming subject to care proceedings, placed in kinship care or removed from the care of mothers who have complex needs, including drug dependence [5–7]. Illicit drug use in the perinatal period raises issues concerning stigma and fear of child removals that arguably do not apply in the same way or to the same extent for alcohol, cannabis, and tobacco use [8]. For this reason, we focused on mapping clinical and practice guidance for the care of women who use or are in treatment for drug use (including illicit and prescribed opioids, stimulants, and benzodiazepines) in the perinatal period, rather than for women who solely use alcohol, cannabis, or tobacco. National Health Service (NHS) maternity services are accessed by almost all pregnant women in the UK, providing an opportunity to monitor and support the health and wellbeing of women and babies through pregnancy, birth, and the postnatal period. Where there are concerns, pregnancy is a key point at which multi-disciplinary teams may come together, to jointly assess and plan for the pregnancy, birth, and future care of the infant [9]. Practitioners across all health and social care services delivering care to pregnant women who use or are in treatment for drug use need clear evidence-based policy and guidance in relation to best practice.

Although policy and guideline documents pertaining to the needs of this population exist internationally, they are often specific to the local, and national context within which they are delivered. This review was primarily concerned with existing guidelines in the UK, although the findings will have relevance to guideline developers and policy makers internationally.

Preliminary searches of Cochrane Library, Joanna Briggs Institute (JBI), Campbell collection and DARE databases suggested that, to date, there has not been a scoping review to map clinical guidance documents currently in use across the UK. Previous reviews, both in the UK and the United States, have focused upon a detailed policy discourse analysis and not provided a general overview of the policy and guideline documents landscape [10–12].

### Objective

To map the landscape of clinical guidelines, treatment protocols and good practice guidance for optimising outcomes and reducing inequalities for women who use or are in treatment for drug use during the perinatal period.

#### Aims

- To identify recommended best practice across health and social care for optimising outcomes and reducing inequalities for women who use or are in treatment for drug use during the perinatal period.
- To identify any gaps in best practice guidelines in relation to the treatment and care needs of women who use, or are in treatment for drug use during the perinatal period.
- To inform the development of a rapid systematic review concerned with the effectiveness of interventions for this population.

#### Definitions

- We used the term women who use or are in treatment for drug use during the perinatal period to refer to our population of concern: women who use illicit and prescribed opioids, stimulants, and benzodiazepines) in the perinatal period, rather than for women who solely use alcohol, cannabis, or tobacco.
- The term domestic abuse is used as it refers to the broad range of abusive behaviours that it might include controlling, coercive, threatening, degrading, violent, or sexually violent behaviour. Perpetrators can be current or ex-partners but can also be other family members or carers. ‘Domestic abuse’ is used in a statutory legislative context, in the UK Government Domestic Abuse Act (2021) [13] and the Domestic Abuse (Scotland) Act 2018 [14], as well as within the majority of the guidance documents included in the review.

## Methods

This scoping review of clinical guidelines and other policy documents aimed to map UK guidelines, treatment protocols and good practice guidance for women who use or are in treatment for drug use during the perinatal period. We endeavoured to identify recommended best practice across health and social care for optimising outcomes and reducing inequalities for these women in the UK, as well as highlight gaps in policy guidance.

This review focussed specifically upon the UK context as it was undertaken as part of a larger NIHR (National Institute for Health Research) funded study (NIHR130619). A core part of this NIHR study involves researchers working with an expert advisory and coproduction group (EACG), including representatives from policy makers, service providers, practitioners across health and social care, and peer researchers.

Scoping review methodology was selected as it allowed us to include and map a variety of documents, creating a descriptive overview of the guidance landscape in the UK pertaining to our topic [15–17]. Scoping reviews were first defined, and their framework outlined by Arskey and O’Malley [16], further developed by Levac et al. [15] and most recently by the JBI methods group [18–20].

“*Scoping reviews are a type of evidence synthesis that aims to systematically identify and map the breadth of evidence available on a particular topic, field, concept, or issue, often irrespective of source (ie, primary research, reviews, non-empirical evidence) within or across particular contexts. Scoping reviews can clarify key concepts/definitions in the literature and identify key characteristics or factors related to a concept, including those related to methodological research.”* [17] ^*[950]*^

This review was conducted following a registered protocol [21], informed by JBI Scoping review guidance [18], and was reported in line with the PRISMA Scr extension [22]. A scoping review differs from systematic review approach as it does not seek to “to present a view regarding the ‘weight’ of evidence in relation to particular interventions or policies” [16]. The purpose of this review was not to define what best practice is but to present the breadth of what was recommended within current guidelines and policy documents and identify any potential gaps in policy provision.

The predefined search strategy aimed to identify key clinical guidelines and other health and social care policy documents relating to women who use or are in treatment for drug use during the perinatal period, and their babies in the UK. This was an iterative process, with policy and guidelines primarily located within the grey literature, it was necessary for our search to extend beyond electronic databases [16]. Our approach to searching is modelled around guidance by Arskey and O Malley [16], and is common to scoping reviews of policy documents [23–25]. Searching was conducted between November 2021 – March 2022 and included:

1. Web-based platforms such as Google Scholar, key government and local authority websites, and organisational and guidance-specific websites (e.g., Royal College of Midwives (RCM); National Institute of Health and Care Excellence (NICE); Scottish Intercollegiate Guidelines Network (SIGN) were searched using identified key words.
2. Electronic database searching (using agreed, database-specific search terms created in consultation with the University of Stirling Health Sciences Librarian, Table 1, and Supplementary Table 1). This was limited to Social Care Online, PsycINFO, CINAHL and Trip, as these were considered most appropriate to capture a broad range of documents, including profession-specific guidance documents.
3. A request was made to all Local Maternity and Neonatal System (LMNS) in England, Wales and Northern Ireland by the London Neonatal Operational Delivery Network via the Operational Delivery Network structure or regional Chief Midwifery Officers.
4. A request for evidence was sent to members of the study EACG as well as other identified UK experts (Supplementary Table 2).
5. The reference list of all included sources of evidence was screened for additional documents.

**Table 1:** Search strategy

**Search Criteria** (Adapted as appropriate for each data base / information source [Supplementary Table 1])	
	(pregnant OR prenatal OR perinatal OR antenatal)
AND	(baby OR infant OR babies OR newborn OR neonate)
AND	(“drug *use” OR “substance *use” OR “drug dependen*” OR “drug treatment” OR opioid OR opiate OR benzo* OR stimulant OR crack OR cocaine OR methadone OR buprenorphine)
AND	(guidelines OR protocols OR “practice guideline” OR “clinical practice guideline” OR policy OR strategy)
AND	(Limit to documents post- 2000)

Identified documents were independently screened by LG, LH, SL, and ES against predefined inclusion and exclusion criteria (Table 2), first by title and abstract / executive summary, and then in full text, with over 25% verified by a second reviewer. Disagreements were resolved by using a third reviewer and/or discussion. A full list of reasons for excluding documents is provided in Supplementary Table 3.

**Table 2:** Inclusion and exclusion criteria

Concept	Include	Exclude
Sources of Evidence	Clinical guidelines, treatment protocols, best practice guidelines, policy documents, written in English and currently in use across the UK, and pertains to any setting (community, hospital, outpatient, prison)	Documents that are not clinical guidelines or other related policy documents
		Documents no longer in use, or have been superseded by a more updated version, or new guidance / policy
		Documents not written in the English language
Participants	Documents about women who are pregnant or within the perinatal period who use or are in treatment for one or more of the following drugs: prescribed opioids (e.g., methadone), illicit opioids (e.g., heroin), benzodiazepines, cocaine/crack, or amphetamines during the perinatal period regardless of age, ethnicity, disability, religious affiliation, cultural identity, gender identity or sexuality	Documents solely about men, babies / children over 1 year old, women not dependent upon or do not use drugs during the perinatal period, babies born to women who do not use or are in treatment for drug use
	Documents about all babies up to one year old born to women who use or are in treatment for drug use in the perinatal period (including illicit and prescribed opioids, stimulants, and benzodiazepines)	Documents solely about women who use other drugs during pregnancy, such as alcohol or tobacco or cannabis, but do not use opiate substances, benzodiazepines, cocaine/crack, or amphetamines
Best practice support / treatment / interventions for women using drugs during the perinatal period for optimising outcomes and reducing inequalities	Documents that report on optimising outcomes for the women using, or in treatment for drug use during the perinatal period and their babies. For example, by improving health and well-being (both physical and mental health)	
	Reducing inequalities – for example, by providing specific support for pregnant women and mothers using drugs in the perinatal period to help them care for their babies, thus reducing the likelihood of babies being removed from their mothers and placed in care (includes all forms of local authority care, kinship care, adoption), or approaches/ interventions specifically addressing poverty and deprivation	

The guidance documents were not assessed for quality, as the purpose of the review was to map what the existing guidelines were, and report upon the suggested practice contained within them. Furthermore, quality assessment is not a prerequisite in scoping review methodology [15, 16, 18].

A predefined data extraction template was used to capture key information about each document. Next, we charted key characteristics (applicability, setting, intended user of the document, relevance, and evidence base) (Table 3), and mapped key best practice recommendations (Table 4). Tables were created during the charting process to organise and present data.

To support charting, categories of recommendations were developed. Researchers LG, LH, SL and ES summarised the main types of recommendations made within a sample of included documents and agreed categories (Table 5). This allowed identification of both commonly made suggestions, and any distinctive or contradictory examples.

### Patient and public involvement

The scoping review protocol, results and findings were shared with the EACG, and their feedback was invited and incorporated into the review. This included sharing initial drafts of the protocol, results of the search and findings with two peer researchers (who have consulted with experts by experience as part of their role in the EACG) who provided constructive feedback around language that was then used to adapt the introduction and initial search criteria. Additionally oral presentations of the protocol, results of the search, and findings of the review were made to the full EACG who supported refinement of our search strategy and suggested informants who may have been able to help identify relevant guidelines / policy documents that were not publicly available. Following presentation of our findings and distribution of a summary report of preliminary findings, discussion amongst EACG members helped to identify potential gaps within the guidelines, such as the provision of mental health support for mothers after their babies have been removed.

## Results

**Fig. 1:**
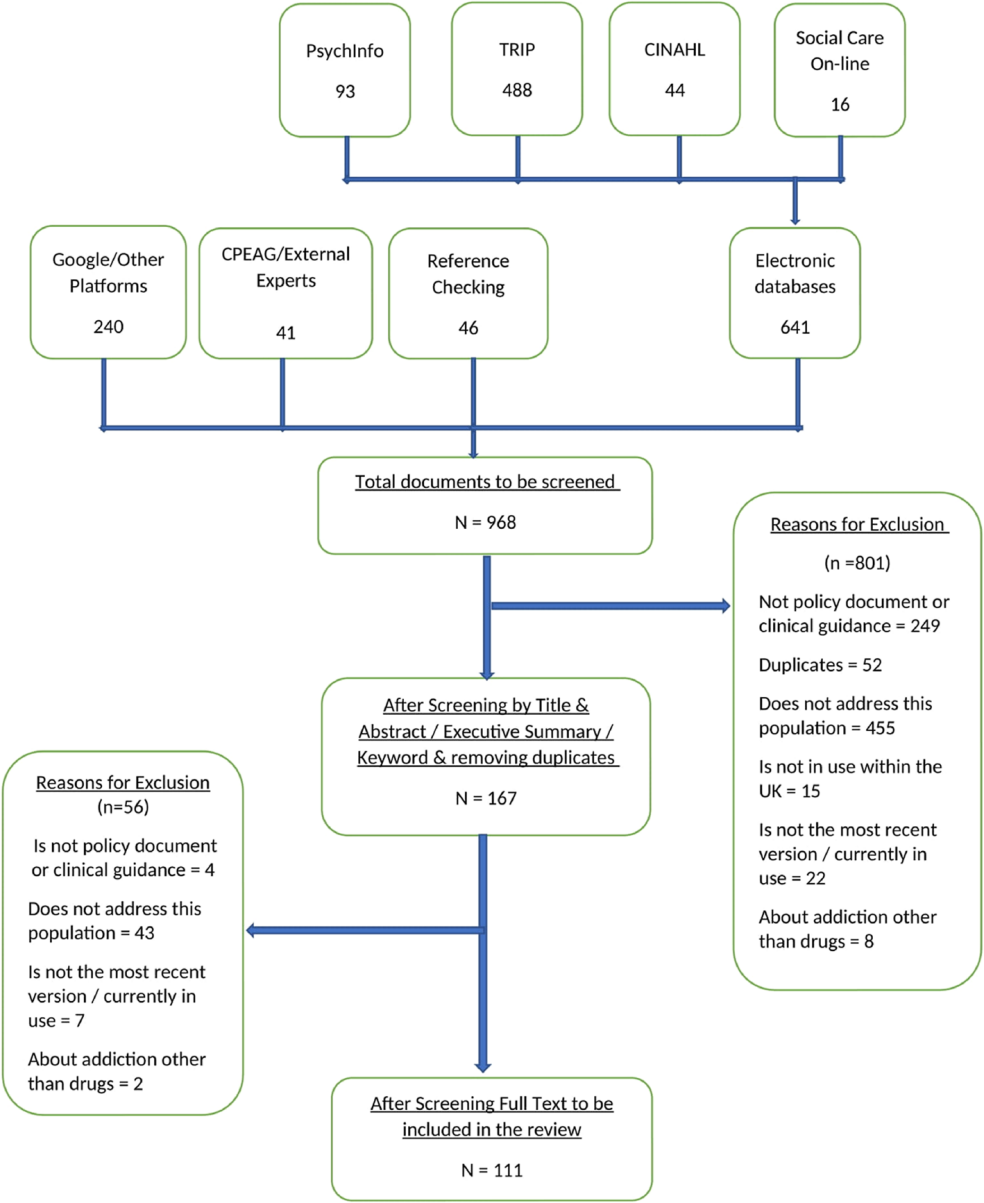
PRISMA diagram

Following screening, a total of 111 guidelines or policy documents published between 2000 and 2022 were included [9, 26–135] (Fig. 1: PRISMA diagram). The following narrative briefly describes the key characteristics of the included documents (which are presented in full in Tables 3 and 4 and Supplementary Tables 4, 5, 6) before summarising the recommendations for identified thematic categories.

**Table 3:** Key characteristics

*Author (Year)*	*Document Title*	*Country*	*Context—Region/Setting*	*User (e.g., midwives)*	*Aim / purpose of document*	*How does document relate to women using drugs in perinatal period?*	*Stage it applies on the perinatal journey*	*Refers to PWWUD as being vulnerable/disadvantaged?*
Greater Glasgow and Clyde NHS (2016)	[CG] Use of alcohol and other drugs in pregnancy: guideline for management flowchart [26]	Scotland	Regional/Greater Glasgow & Clyde/Hospital	Medical (Obs & Gynae, midwives, nurses)	Outlines the medical management of pregnant women who use alcohol and others drugs during pregnancy	Whole document is dedicated to medical management of pregnant women who use alcohol, illicit and prescribed benzodiazepines and opiates	Pregnancy	No
Greater Glasgow and Clyde NHS (2016)	[CG] Use of benzodiazepines in pregnancy. Guidelines for obstetric management [27]	Scotland	Regional/Greater Glasgow & Clyde/Hospital	Medical (Obs & Gynae, midwives, nurses)	Provides guidelines for management and detox from benzodiazepines for pregnant women	Whole document is dedicated to the medical management of detox for women who are pregnant from Benzodiazepines	Pregnancy	No
Greater Glasgow and Clyde NHS (2016)	[CG] Use of Opiates in pregnancy. Guidelines for obstetric management [28]	Scotland	Regional/Greater Glasgow & Clyde/Hospital	Medical (Doctor's prescribing guidance)	Outlines the medical management of women who use opiates while pregnant	Whole document is dedicated to the medical management of pregnant women who use opiates while pregnant	Pregnancy	No
BASW—Hulmes, A. and Galvani, S. (2019)	A child's first 1000 days: the impact of alcohol and other drugs [29]	UK—wide	UK wide / Any setting	Social Workers	Provides guidance for social workers working with parents who use drugs / alcohol within the first 1000 days of a baby’s life (conception up until aged 2 yrs.)	Whole document is relevant and specific to women who use drugs during the perinatal period	Perinatal period	No
Blackpool Better Start. Centre for Early Child Development (2021)	A good practice guide to support the implementation of trauma informed care in the perinatal period [30]	England	Regional/Blackpool/Community & Hospital	All healthcare staff working with perinatal women	Aims to offer additional support for all staff (clinical and non-clinical) working with women in the perinatal period to strengthen trauma informed care practices	Whole document refers to women experiencing trauma during the perinatal period and acknowledges that substance using women may experience trauma	Perinatal period	Yes
Department of Health, Social Services and Public Safety NI (2012)	A Strategy for Maternity Care in Northern Ireland 2012–2018 [31]	Northern Ireland	National / Maternity services—Community & Hospital	Maternity care providers—Nursing, Midwifery & Allied health services	Document outlines strategic direction and clear objectives for maternity care provision in Northern Ireland. 6	Document is applicable as universal for all pregnant women but also lists pregnant women who use drugs as a category at higher risk of health inequalities, and of high-risk pregnancies. There are fleeting specific references to their care throughout, and to the NICE guidelines CCG 110	Perinatal period	Yes
University Hospitals Birmingham NHS Foundation Trust (2019)	Abstinence Syndrome [32]	England	Regional/Birmingham/Hospital	Medical (Neonatal medical, midwifery and nursing staff and staff on postnatal wards)	To outline information regarding the management of infants at risk of neonatal abstinence syndrome	The whole document is relevant as it refers to the care of infants who have developed NAS, and by association covers PWWUD	Postnatal period	No
NHS Orkney & Orkney Island Council (2020)	Additional support pathway for women with vulnerabilities [33]	Scotland	Regional / Orkney / Community & Hospital	Midwives	Guidance for midwives around identifying, assessing, supporting, and managing unborn babies where there are identified risk factors / concerns	The whole document is relevant as drug use is listed as a complex medical factor which can impact vulnerability for mother and baby	Pregnancy	Yes
NICE (2014; 2020)	Antenatal and postnatal mental health: clinical management and service guidance [34]	UK—wide	National / Hospital	Healthcare professionals, Commissioners, Social services, Voluntary and private sectors	This guideline covers recognising, assessing, and treating mental health problems in women who are planning to have a baby, are pregnant, or have had a baby or been pregnant in the past year	The document contains a relevant section on alcohol and drug misuse in pregnancy (pg.36)	Perinatal period	No
NICE (2021)	Antenatal Care [35]	UK—wide	National / Any setting	Healthcare Professionals, Commissioners, and providers of maternity care	This guideline covers the routine antenatal care that women and their babies should receive. It aims to ensure that pregnant women are offered regular check-ups, information, and support	It is applicable as a universal document for all pregnant women. In relation to the specific care needs of pregnant women using drugs it refers to the NICE guidance on Pregnancy and complex social factors	Pregnancy	Yes
Royal College of Obstetricians and Gynaecologists (2012)	Bacterial Sepsis Following Pregnancy. Green–top Guideline No. 64b, [36]	UK—wide	National / Hospital	Medical (Doctors, clinicians and healthcare professionals working with pregnant women)	To provide guidance for obstetricians, gynaecologists, and related medical professionals on the management of sepsis in the puerperium	Document contains a relevant section related to PWWUD and links to bacterial sepsis (pg6)	Postnatal period	No
Lingford-Hughes, Welch, Peters and Nutt (2012)	BAP updated guidelines: evidence-based guidelines for the pharmacological management of substance abuse, harmful use, addiction and comorbidity: recommendations from BAP [37]	UK—wide	National / Any setting	Pharmacists	This guideline relates to the pharmacological management of withdrawal, short- and long-term substitution, maintenance of abstinence and prevention of complications, where appropriate, for substance abuse or harmful use or addiction as well management in pregnancy, comorbidity with psychiatric disorders and in younger and older people	There is a specific section on the pharmacological management of pregnant women and recommended practice for the assessment/antenatal care, opioids, methadone, buprenorphine, slow-release oral morphine, detoxification, and stimulants	Pregnancy	No
Bristol, North Somerset and South Gloucestershire Clinical Commissioning Group (2021)	Benzodiazepines and Z-drugs as Hypnotics and Anxiolytics [38]	England	Regional/Bristol / Any setting	Medical (clinicians and local practitioners)	A support document consolidating national guidance, expert opinion, and local resources to aid local practice including prescribing, de-prescribing/withdrawal and self-care	Contains a relevant section to PWWUD on prescribing benzodiazepines	Perinatal period	No
Public Health England (2017)	Better care for people with co-occurring mental health and alcohol/drug use conditions [39]	England	National / Any setting	Commissioners and service providers of mental health and drug treatment services	Guidance for service providers and commissioners to inform provision of services for people with co-occurring mental health and alcohol / drug use conditions	PWWUD or have recently given birth and have a co-occurring mental health condition are recognised as a vulnerable group, and the document contains specific as well as generic practice recommendations for them	Perinatal period	Yes
Care Quality Commission (2018, reviewed 2019)	Brief guide: Substance misuse services – People in vulnerable circumstances [40]	England	National/Community	All health and social care providers, practitioners, and service inspectors	Outlines that people using substance misuse services may need extra support temporarily or long-term either because of their personal circumstances, the health conditions they have, or other needs and complexities	Whole document is relevant, and includes specific sections—PWWUD listed as a specific population who may be vulnerable to risk, includes specific mention of risk to mother and child on p.5	Perinatal period	Yes
British Association for Psychopharmacology (2017)	British Association for Psychopharmacology consensus guidance on the use of psychotropic medication preconception, in pregnancy and postpartum 2017 [41]	UK—wide	National / Specific Professions /Any setting	Healthcare professionals (e.g., neurologists, psychiatrists)	Guidance around the use of psychotropic medication in pregnancy and post-partum	Specific suggestions are made in relation to prescribing medications for PWWUD	Pregnancy	No
Change, Grow, Live (2019)	Change, Grow, Live (CGL) Procedure: Substance Misuse in Pregnancy [42]	UK—wide	Organisational/Hospital & Community	Healthcare professionals who work with PWWUD (CGL permanent and temporary staff, volunteers and sub-contracted agencies involved in delivering or supporting services offered by the organisation)	This procedure has been produced to provide information to health care professionals involved in the care of pregnant women who have drug problems, to enable them to provide appropriate care and advice	Whole document is relevant and outlines care of PWWUD	Perinatal period	Yes
Aberdeen Alcohol & Drugs Partnership (2019)	Charter 3.2 Births affected by drugs (Health improvement plan) [43]	Scotland	Regional / Aberdeen/ Any setting	Community Planning Aberdeen Partnership members & Aberdeen Drug & Alcohol Partnership members (Local statutory & non-statutory service providers)	To reduce the number of births in Aberdeen affected by drugs by 0.6%, by 2022	Whole document is relevant to PWWUD	Pregnancy	Yes
NICE (2017)	Child abuse and neglect [44]	UK—wide	National/Hospital & Community	All practitioners who encounter children & young people, including commissioners, managers and risk assessment practitioners	Aims to help anyone whose work brings them into contact with children and young people to spot signs of abuse and neglect and to know how to respond	Document contains some mentions of PWWUD in relation to child abuse and neglect	Perinatal period	No
Outer Hebrides Drug and Alcohol Partnership and Outer Hebrides Child Protection Committee (2018)	Children affected by parental drug or alcohol related problems GIRFEC oriented inter-agency guidelines [45]	Scotland	Regional / Outer Hebrides / Any setting	Any agency or professional working with children	To provide clarity about what is expected of staff working with children, and who does what within an interagency context. They must be used together within the context of Outer Hebrides Inter-agency Child Protection Procedures (2015). For staff working with adults these are supplementary to the Single Shared Assessment	Whole document is relevant to PWWUD as pregnancy is identified as a critical period, and they are considered with the context of children living with parents who use substances problematically. There is also a specific section dedicated to the "Management of pregnant substance users"	Perinatal period	Unclear
HIPS Safeguarding Children Partnership (2022)	Children living in households where there is substance misuse [46]	England	Regional / Hampshire, Isle of Wight, Portsmouth and Southampton / Any setting	All staff and agencies working with children, and families; all local authorities, clinical commissioning groups, police and all other organisations and agencies	Specific local safeguarding protocol for children where parents misuse substances	There is a specific section about substance misuse in pregnancy	Perinatal period	Unclear
Hull Safeguarding Children Partnership (2022)	Children of parents or carers who misuse substances [47]	England	Regional / Hull / Any setting	Community Services / Members associated with Hull Safeguarding Children's Partnership	Guidance for Hull Safeguarding Partnership which outlines procedure dealing with children of substance using parents	Document contains a specific section on substance misuse under 'Risks'	Perinatal period	Yes
Regional Child Protection Procedures for West Midlands (2022)	Children of parents who misuse substances [48]	England	Regional / West Midlands / Any setting	Any agency or professional working with women who use drugs, parents and or children	Specific local child protection procedures where parents use substances	There are specific references throughout to pregnant women who use substances	Perinatal period	Yes
South Lanarkshire Partnership (2021)	Children's Service Plan: 2021–2023 [49]	Scotland	Regional / South Lanarkshire / Hospital & Community	Partnership members—Medical/Local NHS/Community/Police + Fire etc	Regional plan for children’s services in South Lanarkshire	The document contains relevant mentions children and young people affected by substance use in pregnancy remaining an area of need (p.26)	Perinatal period	Unclear
HM Prison Service (2000)	Clinical services for substance users [50]	UK—wide	National / Specific Profession / Prison	Staff working in prison	Outlines evidence-based standards for effective clinical management of people for "substance misusers"	This document applies to PWWUD and are in prison as universal document but also contains specific recommendations regarding local policy provision within each prison as well as in relation to their treatment and care	Perinatal period	No
NICE (2016)	Coexisting severe mental illness and substance misuse: community health and social care services [51]	UK—wide	National / Hospital & Community	Community and mental health service commissioners and providers/Primary care providers/Staff in the criminal justice system	The document covers how to improve services for people aged 14 and above who have been diagnosed as having coexisting severe mental illness and substance misuse. It aims to provide a range of coordinated services that address people's wider health and social care needs, as well as other peripheral challenges such as employment and housing	The document contains relevant mentions to the care of the population and their mental health care—identifies PWWUD as a particularly vulnerable group	Pregnancy	Yes
Derbyshire Safeguarding Children Board (2020)	Derby and Derbyshire Multi Agency Protocol for Pre-Birth Assessments and Interventions [52]	England	Regional/ Derbyshire / Any Setting	Any agency or professional working with pregnant women and their families	Safeguarding protocol around pre-birth assessment where there are concerns identified about the unborn baby’s well-being	Whole document is relevant as PWWUD are identified as they are identified as vulnerable group potentially in need of early help. There is also a specific section for "Parents with Substance (drugs and /or alcohol) Issues", and recommended actions to be taken in their assessment and support	Pregnancy	No
The Royal College of Psychiatrists and the Faculty of Forensic and Legal Medicine (2020)	Detainees with substance use disorders in police custody: Guidelines for clinical management [53]	UK—wide	National/Specific Profession/Police stations	Healthcare professionals working in the field of forensic and legal medicine	Guidelines developed for staff (including healthcare practitioners) who work with detainees in custody with substance use problems	Document includes a specific Sect. (2.2.4) on pregnant detainees	Pregnancy	Unclear
Department of Health -Clinical Guidelines on Drug Misuse and Dependence Update 2017 Independent Expert Working Group (2017)	Drug misuse and dependence UK guidelines on clinical management [54]	UK—wide	National / Any setting	Healthcare professionals, Providers and commissioners of treatment for people who misuse or are dependent on drugs	Provides guidance for clinicians prescribing and / or delivering drug treatment for people who misuse or are dependent on drugs	The document applies to PWWUD as they are people who misuse or are dependent on drugs. There is also a specific section on pregnancy and neonatal care	Perinatal period	No
Doncaster and Bassetlaw Teaching Hospitals NHS Foundation Trust (2017)	Drug Misuse Management in the Acute Hospital Setting – guidelines [55]	England	Regional/Doncaster and Bassetlaw/Hospital	Medical/Nursing staff	To act as a resource in the management of patients with drug misuse issues, how to deal with common problems that arise and how to signpost / refer to community treatment providers	Document includes a Sect. (11.3) refers to the care of PWWUD	Pregnancy	No
National Collaborating Centre for Mental Health (2019)	Drug misuse: Opioid detoxification The NICE Guideline [56]	UK—wide	National / Any setting	Clinicians and service commissioners	To provide guidance on the clinical management of opioid detoxification	There are specific references throughout to pregnant women who use substances	Perinatal period	Unclear
NHS (2021)	Equity and equality Guidance for local maternity systems [57]	UK—wide	National / Hospital & Community	Maternity care service providers and practitioners	This guidance seeks to respond to the findings of MBRRACE-UK report which found worse maternal and perinatal mortality outcomes for those from Black, Asian and Mixed ethnic groups and those living in the most deprived areas	The document covers relevant management of women with complex social factors (p.19), and PWWUD are identified as a category at risk of extreme disadvantage	Pregnancy	Yes
NHS Lothian (2021)	Expressed breast milk: Information for carers of vulnerable babies [58]	Scotland	Regional / Lothian / Community	Carers of vulnerable babies	Guidance for carers of vulnerable babies regarding breastfeeding	Document is relevant to babies born to PWWUD, whose babies may be cared for by someone else. There is also a specific section on substance misuse and breastfeeding	Perinatal period	No
Scottish Government (2021)	Families Affected by Drug and Alcohol Use in Scotland: A Framework for Holistic Whole Family Approaches and Family Inclusive Practice [59]	Scotland	National/Community	Commissioners/Service delivery agencies /partnerships which have a collective leadership role in relation to alcohol and drug related harms	Provides a framework, in line with other national drug/alcohol strategies and linked policy initiatives for the development and delivery of a consistent approach for families affected by substance use across Scotland	The document is universal and is relevant as it relates to families affected by drug and alcohol use	Perinatal period	Yes
North Lanarkshire CPC & South Lanarkshire CPC & Lanarkshire ADP (2015)	Getting it Right for Children and Families Affected by Parental Alcohol and Drug Use in Lanarkshire [60]	Scotland	Regional / Lanarkshire / Any setting	All practitioners working with children and families affected by problematic parental drug / alcohol use	Good practice framework for all practitioners working with children and families affected by parental drug / alcohol use	Whole document is relevant, but there are also specific references to PWWUD, and sections on maternity and neonatal care, and pre-birth child protection case conferences	Perinatal period	Yes
(ELBEG-PP 2013) Whittaker, A., Templeton, L., Mitchell, F., Hill, L. & Neilson, A. (2013)	Getting it right for children and families affected by parental problem alcohol and drug use: Guidelines for agencies in Edinburgh and the Lothians [61]	Scotland	Regional/The Lothians/Hospital & Community	Medical/Community/Service delivery—agencies who may work with families where this is drug misuse	Provides an operational framework applicable to all statutory and third sector agencies and practitioners to ensure that they work together to promote the welfare of, and to safeguard children. It outlines guidelines for staff and agencies in relation to screening, assessment, information sharing, support and intervention for all children and parents, including expectant parents	The document contains a relevant section related to the multiagency guidance and procedure for children and unborn babies of pregnant women who use drugs (Sect. 9)	Perinatal period	Yes
Scottish Government (2013)	Getting Our Priorities Right (GOPR) [62]	Scotland	National / Any setting	All practitioners working with children and families affected by problematic parental drug / alcohol use	To provide an updated good practice framework for all child and adult service practitioners working with children and families affected by problem parental alcohol and/or drug use	The whole document is relevant to PWWUD, mothers who use drugs and their babies, however there are also a few specific references to best practice procedures for this population	Perinatal period	Yes
Forth Valley Drug Partnership and Child Protection Committee (2019)	Getting Our Priorities Right for Children and Families affected by Parental Alcohol and Drug Use: Guidance from the Forth Valley Alcohol and Drug Partnerships and Child Protection Committees [63]	Scotland	Regional/Forth Valley/Hospital & Community	Medical/Community/Service delivery—agencies who may work with families where this is drug misuse	Developed to support practitioners and managers in their work with adults, children, young people and families affected by problematic parental drug and/or alcohol use. It is aimed at those working in children and adult services within the public, private and third sector agencies across Forth Valley	The document contains a specific section—4a) Pregnancy and the unborn child (pg. 33) which contains "Key messages from National Guidance"	Perinatal period	Unclear
East Ayrshire Child Protection Meeting (2014)	Good Practice—Working with Pregnant Women with Parental Substance Misuse [64]	Scotland	Regional/ East Ayrshire/ Any setting	Child Protection committee members and practitioners working with women who are referred on vulnerable pregnancy care pathway	"To provide East Ayrshire Child Protection Committee (EACPC) and other relevant individuals with a rationale relating to the effectiveness of interventions in improving outcomes for problematic substance use. To support practitioners in evidence-based practice to inform work with women referred under the High-Risk Pregnancy protocol due to substance misuse in pregnancy."	The whole document is specific to PWWUD	Perinatal period	Unclear
Department of Health, Social Services, and Public Safety (2020)	Guidance for Alcohol and Drug services in Northern Ireland to best deliver treatment and care during the COVID-19 pandemic [65]	Northern Ireland	National/Hospital & Community	Practitioners working in alcohol and drug services	Developed during the beginning of the Covid-19 outbreak/lockdowns, the guidance, aimed at Alcohol and Drugs services in Northern Ireland, outlines the how care and services will continue and adapt during Covid-19	The document contains mentions of PWWUD and their vulnerability to Covid-19	Pregnancy	No
NHS Grampian (2019)	Guidance for the use of buprenorphine products for the treatment of opioid dependence in NHS Grampian [66]	Scotland	Regional / Grampian / Any setting	Practitioners involved in prescribing buprenorphine	Outlines procedure for prescribing buprenorphine products for managing opioid dependence	Document contains specific sections on the use of burenorphine during pregnancy and breastfeeding	Perinatal period	No
Barnsley Safeguarding Children Partnership (2022)	Guidelines for multi-agency assessment of pregnant women and their babies in cases where there is substance misuse [67]	England	Regional/Barnsley/Hospital & Community	All practitioners working to safeguard children (inc. police, healthcare practitioners, local authority and third sector)	These guidelines encourage early uptake and normalising of antenatal care for substance using women whilst signposting relevant additional services, the establishing of an action plan to meet the needs of the pregnant women and her family (including additional children), and that communication exist between all engaged professionals to ensure concerns are dealt with appropriately	Whole document is relevant, relating to the multi-agency care of pregnant women who use drugs	Perinatal period	No
Leeds City Council (2010)	Guidelines for the assessment of parental substance misuse [68]	England	Regional / Leeds / Community	Social care workers	Outlines procedures for social care staff working with families where there is parental substance misuse	Whole document applies to PWWUD and mothers who use drugs. There are also specific recommendations made for this population	Perinatal period	Unclear
St Mungo's (2017)	Homeless Pregnancy Toolkit [69]	UK—wide	National/Community	Staff working with homeless pregnant women	To improve outcomes for homeless pregnant women and improve health and development outcomes for babies born to homeless women by creating equitable quality of service and consistency of approach for all pregnant homeless women. It also aims to implement good practice for homeless women with complex social needs	Whole document is relevant, and includes a specific section on Substance Use (pg.6)	Perinatal period	Yes
NHS (2017)	Implementing Better Births, a resource pack for local maternity systems [70]	England	National / Any setting	Maternity Services	Guidance for all maternity services to implement vision set out in Better Births document	Whole document is relevant as universal to maternity care services. There is only one specific mention of pregnant women who use drugs	Perinatal period	No
Glasgow Child Protection Committee (2008)	Inter-Agency procedural guidance for vulnerable women during pregnancy [71]	Scotland	Regional / Glasgow / Any setting	Any agency or professional working with vulnerable pregnant women and their families	Child protection guidance to support vulnerable parents to ensure their child's safety	Whole document is relevant and specific to women who use drugs during the perinatal period, as substance misuse is identified as factor attributed to vulnerability in pregnancy	Perinatal period	Yes
NICE (2014; 2017)	Intrapartum care for healthy women and babies [72]	UK—wide	National/Hospital	Healthcare Professionals, Commissioners and providers of maternity care	The guidance the care of healthy women and their babies, during labour and immediately after the birth, and helps women to make an informed choice about where to have their baby. The document also aims to reduce variation in areas of care such as foetal monitoring during labour and management of the third stage of labour	The document mentions women who use drugs recreationally, and their care needs	Pregnancy	No
NICE (2019)	Intrapartum care for women with existing medical conditions or obstetric complications and their babies [9]	UK—wide	National / Any setting	Obstetricians, midwives, anaesthetists and other healthcare professionals caring for women in labour; Providers and commissioners of maternity services; Pregnant women, their families and carers	Clinical guidance on care during labour and birth for women who need extra support because they have a medical condition or complications in their current or previous pregnancy, and women who have had no antenatal care	The whole document is relevant for some PWWUD	Perinatal period	Yes
Wilson, C., Boxhall, C. and Kelleher, M. (2019)	Lambeth drug and alcohol service guidelines for the management of substance misuse in the perinatal period [73]	England	Regional/Lambeth/Any setting	Maternity and addiction service providers	The guidelines aim to facilitate a coordinated approach to supporting women misusing substances and their families in the perinatal period. It places focus on joint working between maternity and addictions services, alongside other agencies	The whole document is relevant as it refers to the management of addictions during the perinatal period (inc. substances/opioids, alcohol and tobacco)	Perinatal period	Yes
Royal College of Obstetrics and Gynaecology (2010; 2017)	Late intrauterine foetal death and stillbirth [74]	UK—wide	National / Any setting	Obstetricians and midwives	To identify evidence-based options for women (and their relatives) who have a late intrauterine foetal death (IUFD): after 24 completed weeks of pregnancy) of a singleton foetus, and provide guidance on care before, during and after birth, and care in future pregnancies	The whole document is universally relevant if a PWWUD has an IUFD. There are also specific to references to testing the foetus for indications of substance misuse	Perinatal period	No
NICE (2021)	Looked-after children and young people [75]	UK—wide	National/ Any setting	All social, health and education practitioners looking after children including commissioners, managers and policymakers	The guidance document outlines how organisations, practitioners and carers should work together to deliver high-quality care, stable placements and nurturing relationships for looked-after children and young people	The document includes references to PWWUD in relation to child and family reunification proceedings	Perinatal period	No
University Hospitals Birmingham NHS Foundation Trust (2020)	Management of substance misuse in pregnancy [76]	England	Regional/Birmingham/Hospital	All staff working within maternity (Obstetrics, Midwifery, Anaesthetics, Neonatologists)	The document's aim is to reduce maternal and neonatal morbidity and mortality associated with substance misuse in pregnancy. It outlines the implications of substance misuse in pregnancy, both for mother and baby, appropriate referral criteria and care pathways and discharge planning	The whole document is relevant; it covers the care of PWWUD from the antenatal period to discharge and follow-up appointments. It includes management of specific substances, and the care pathways and referrals necessary for midwives managing PWWUD	Perinatal period	Yes
University Hospitals Plymouth (2019)	Management of substance use during pregnancy and the postnatal period [77]	England	Regional / Plymouth/ Hospital	Healthcare practitioners	Provide guidance for healthcare practitioners in relation to their role and responsibilities to ensure they provide appropriate care and know who to refer on to / seek guidance from	The whole document is specific to PWWUD	Perinatal period	Yes
Royal College of Obstetricians and Gynaecologists (2011)	Management of women with mental health issues during pregnancy and the postnatal period [78]	UK—wide	National/Specific Profession/Hospital	Obstetricians and midwives	Aims to highlight the role of maternity services in the early identification of high-risk women and assessment of current mental illness and describes principles of service organisation for health providers to meet these needs	The document includes recommendations for discussing drug use in pregnant women, and subsequent care	Perinatal period	Unclear
NHS York Teaching Hospital (2019)	Maternity services guideline: Antenatal appointments guideline [79]	England	Regional / York / Community	Maternity health care professionals	Provide a framework "to enable the consistent provision of high quality, evidence based holistic care to pregnant women" accessing the Trust maternity services	Is applicable as a universal document for all pregnant women, but also identifies women who misuse drugs during pregnancy in the 'high-risk' category	Perinatal period	No
Evelina London and NHS Guy's and St Thomas' NHS Foundation Trust (2021)	Maternity substance misuse in pregnancy guideline [80]	England	Regional/ London/ Hospital	Obstetricians and midwives	Provides guidance on the management of substance misuse in pregnancy and the immediate post birth period	The whole document is relevant as it outlines the care of PWWUD and their babies from Antenatal booking appointments to discharge. It refers to in-hospital/in-patient care and hospital-based appointments	Perinatal period	No
Wolverhampton Safeguarding Children Board (2013)	Multi-Agency Guidance Hidden Harm – Parental substance misuse and the effects on children [81]	England	Regional / Wolverhampton / Any setting	Practitioners working with children or families where parental substance misuse is an issue	Guidelines for multi-agency practitioners in Wolverhampton working with children, young people and families and/or adults who have care of children where substance misuse is a factor which affects their lives. To provide: information on substance misuse and how this may impact upon an individual’s ability to care for a child, a structure for communication and collaborative working, and a risk management tool. "The purpose of this practice guidance is to assist staff in all agencies in identifying situations where action is needed to safeguard a child and promote their welfare as a result of their parent’s drug and / or alcohol use."	The whole document is relevant to PWWUD, mothers who use drugs and their babies, and there is a specific section on "Pregnancy and the Unborn Baby"	Perinatal Period	Yes
Scottish Government (2021)	National Guidance for Child Protection in Scotland 2021 [82]	Scotland	National/ Any setting	All staff working with children and families	Outlines the expectations and responsibilities for those engaged in protecting children and will support the care and protection of children	The document contains mentions of PWWUD and their children in relation to care proceedings and safeguarding	Perinatal period	Yes
Highland Council & NHS Highland (2020)	North Highland Vulnerable Pregnancy Pathway—Taking a trauma informed approach in understanding and responding to vulnerability in pregnancy [83]	Scotland	Regional / North Highland / Community	All staff in agencies working with vulnerable pregnant women	"The purpose of this protocol is to ensure standardised timely and proportionate care is received by all vulnerable women and their families across North Highland. It aims to provide clear guidance for staff around roles and responsibilities and expected timescales for those who may need multi agency support. "(2)	This document is relevant as is universal to all pregnancies identified as vulnerable—it has a few specific references to pregnant women who use substances throughout and refers to another guideline for this population	Pregnancy	No
Staffordshire, Shropshire & Black Country Newborn and Maternity Network and Southern West Midlands Maternity and Newborn Network (2017)	Obstetric Guidelines 2017–19 [84]	England	Regional/The Midlands/ Hospital	All staff in obstetric management	The document functions as guidance for all staff working in obstetric management, and aims to create a more uniform standard of care across the Staffordshire, Shropshire & Black Country and Southern West Midlands Newborn and Maternity Networks’ hospitals	The document contains references to PWWUD in relation to antenatal care, mental health care during pregnancy and in postnatal care	Perinatal period	Yes
NICE (2022)	Opioid dependence: Scenario: Managing special circumstances [85]	UK—wide	National / Any setting	Clinicians prescribing treatment for opioid dependency	Provides specific guiding on managing opioid dependency in specific circumstances	The document contains a relevant section on opioid management in pregnancy	Perinatal period	No
Orkney Health and Care (2021)	Orkney Alcohol and Drugs Partnership Strategy 2021–31 [86]	Scotland	Regional/ Orkney/ Any setting	All practitioners working with children and families affected by problematic parental drug / alcohol use	The strategy outlines the vision, outcomes and approach to delivery of the Orkney Alcohol and Drugs Strategy and considers associated local and national policy to reduce drug and alcohol related harm	The document contains mentions of PWWUD and family care	Perinatal period	No
Outer Hebrides Community Planning Partnership (2020)	Outer Hebrides integrated children's services plan [87]	Scotland	Regional/Outer Hebrides / Any setting	All practitioners working with children, young people and families	The Integrated Services Plan outlines the joint vision, shared priorities and the common outcomes the partnership aims to achieve for children, young people and families in the Western Isles. It is based on the statutory guidance Children and Young People (Scotland) Act 2014 and the subsequent Statutory Guidance on Part 3: Children’s Services Planning – Second Edition 2020, and covers all agencies, professions and staff working with children, young people and families	The document The document contains references to support systems in place for women who use drugs, and identifies these as a vulnerable group, with a focus on supporting substance misusing mothers, their children and young people and child safeguarding and protection	Perinatal period	Yes
The Royal College of Midwives (2020)	Parental emotional wellbeing and infant development [88]	UK—wide	Specific profession / Community & Hospital	Midwives	Provides "information and advice on the inextricably linked issues of parental mental health, the parent-baby relationship, and infant development"	There is a specific section on alcohol and drug use during pregnancy	Perinatal period	Unclear
Milton Keynes Inter-Agency Safeguarding Children Procedures (2022)	Parental Substance Misuse [89]	England	Regional/ Milton Keynes/ Any setting	Social care workers	This is safeguarding guidance aimed at professionals who come across children, including unborn babies, who have parents who use drugs	This document includes a relevant section on safeguarding procedures for PWWUD	Perinatal period	No
Public Health England (2021)	Parents with alcohol and drug problems: adult treatment and children and family services [90]	England	National / Any setting	Directors of public health and commissioners and providers of adult alcohol and drug treatment and children and family services	Guideline "outlines the main issues for families affected by parental alcohol and drug problems and shows how services can work together to support them."	Applies as universal guidance around parents using drugs, only a few specific references to pregnant women	Perinatal period	Unclear
Scottish Government (2020)	Perinatal & Infant Mental Health Programme Board 2020–2021 Delivery Plan [91]	Scotland	National/ Any setting	All practitioners working in perinatal and infant mental health	The program is working towards "perinatal and infant mental health services that are responsive, timely and address the changing needs of women and families throughout pregnancy and the early years of life."	The document contains references to PWWUD in relation to supporting mental health in the perinatal period	Perinatal period	No
Sussex Partnership NHS Foundation Trust (2018)	Perinatal Mental Health: Prescribing guidance for trust prescribers and GPs [92]	England	Regional / Sussex / Community	G. P’s and other prescribing practitioners	Provide guidance to practitioners prescribing medications to women during pregnancy for mental health issues	There is a section dedicated to "medications for substance misuse"	Pregnancy	No
NICE (2021)	Postnatal Care [93]	UK—wide	National/ Hospital	Healthcare Professionals, Commissioners and providers of maternity care	This guidance document outlines the routine postnatal care that women and their babies should receive in the first 8 weeks after the birth	This is universal guidance, and contains some relevant recommendations related to the postnatal care of women who use drugs	Postnatal period	No
Dumfries and Galloway—– Strategic Pre-Birth Planning Group (2019)	Pre-birth assessment protocol for vulnerable pregnancies [94]	Scotland	Regional / Dumfries & Galloway / Community & Hospital	All agencies / practitioners providing care to pregnant women and their families	Aim of the protocol: "The overall aim of this protocol is to support professionals in undertaking holistic, needs led / person centred assessments for vulnerable pregnant women and unborn babies that will identify risks and lead to a timely, proportionate and appropriate response to minimise any risk factors / vulnerabilities identified."	Problematic substance misuse in pregnancy is identified as being a vulnerable pregnancy, and therefore the document is relevant to this population. There are also specific references and recommended treatment / actions for PWWUD	Pregnancy	Yes
NICE (2010; 2018)	Pregnancy and complex social factors: a model for service provision for pregnant women with complex social factors [95]	UK—wide	National/ Hospital	Healthcare Professionals, Commissioners and providers of maternity care	This guideline sets out what healthcare professionals as individuals, and antenatal services, can do to address needs and improve pregnancy outcomes for vulnerable women	Guideline applies to all pregnant women with complex social factors; women who misuse substances are identified as one of four exemplar groups	Pregnancy	Yes
NHS Lothian (2011)	Pregnancy and problem substance use [96]	Scotland	Regional / Lothian / Community	G. P’s	To provide guidance to G. P’s on providing care to women who use drugs during pregnancy	Whole document is specific to PWWUD	Perinatal period	No
NHS Lothian Quality Prescribing Group Substance Misuse Directorate (2016)	Pregnancy Guidance [97]	Scotland	Regional/ Lothian/ Any setting	Substance misuse staff	The guidelines are to be used by substance misuse staff treating pregnant women with drug and alcohol problems	The whole document is relevant as it refers to the management and care of pregnant women who use drugs	Perinatal period	Yes
Ministry of Justice and HM Prison and Probation Service (2021)	Pregnancy, mother and baby units (MBUs), and maternal separation from children up to the age of two in women’s prisons [98]	UK—wide	Specific profession / Prisons	Prison staff	An operational policy for mother and baby units in prisons	Universal document for all pregnant women and babies in prison, but also has specific sections on pregnancy and women who use drugs	Perinatal period	Yes
Camden and Islington NHS Foundation Trust (2019)	Prescribing guidance for substance misuse services. [99]	England	Regional/ Camden and Islington/ Any setting	Healthcare practitioners working in substance misuse prescribing	Prescribing guidelines for workers in substance use services to promote evidence-based prescribing in line with national guidance (NICE and PHE)	The document contains a relevant section on prescribing guidance for pregnant opiate dependent clients	Perinatal period	Yes
Department of Health, Social Services, and Public Safety (2020)	Preventing Harm, Empowering Recovery: A strategic framework to tackle the harm from substance use (2021–31) [100]	Northern Ireland	National / Any setting	Policy makers, service commissioners, service providers, practitioners, the public	National drugs strategy—includes aim to reduce stigma, increase access to and provision of high-quality treatment. Is outcome based	Universal document applicable to all who use substances—however they also identify high risk groups and name "vulnerable women and individuals in the pre- and post-natal period;" as one of these groups	Perinatal period	Yes
Royal College of Obstetricians and Gynaecologists (2015)	Reducing the Risk of Venous Thromboembolism during Pregnancy and the Puerperium (2015) [101]	UK—wide	National/ Hospital	Obstetricians and midwives	Aims to provide clinical, evidence-based advice on the prevention of venous thromboembolism (VTE) during pregnancy, birth and following delivery. It subsequently aims to reduce maternal deaths	The document contains specific recommendations and risk assessment procedure for pregnant substance using women	Pregnancy	No
Scottish Government (2018)	Rights, Respect and Recovery Scotland’s strategy to improve health by preventing and reducing alcohol and drug use, harm and related deaths [102]	Scotland	National / Any setting	Policy makers, service commissioners, statutory and non-statutory service providers and practitioners, people who use drugs and their families	Provides guidance on best approach to supporting people with drug / alcohol issues and aims to "reduce the use of and harm from alcohol and drugs, with a particular focus on reducing alcohol and drug deaths."	Universal and applicable to all people who use drugs but also contains a specific section on maternal and infant health	Perinatal period	Unclear
Public Health England (2018)	Safeguarding and promoting the welfare of children affected by parental alcohol and drug use: a guide for local authorities [103]	England	National/Any setting	Local authorities (commissioners) and substance misuse services	"A guide for local authorities and substance misuse services to help them work together to safeguard and promote the welfare of children."	The document covers the safeguarding of children in family settings, and contains recommendations for PWWUD	Perinatal Period	No
Rotherham Safeguarding Children Partnership (2015)	Safeguarding Children of Drug Misusing Parents [104]	England	Regional /Any setting	All practitioners working with children and families affected by problematic parental drug / alcohol use	Local safeguarding procedure manual advice for professionals where a parent is using drugs	Specific references to PWWUD throughout the document	Perinatal period	Unclear
Cumbria, Northumberland Tyne and Wear NHS Foundation Trust (2019)	Safeguarding Children Practice Guidance Note Addiction Services—Pregnancy Pathway and Guidance – V02. [105]	England	Regional/ Cumbria, Northumberland, Tyne and Wear/Any setting	Substance misuse staff	Outlines the role of Cumbria Northumberland, Tyne and Wear NHS Foundation Trust (the Trust/CNTW) Substance Misuse Services and their procedure for working with women engaging in substance misuse services whilst pregnant	The whole document is relevant, focussing on pregnant women who use drugs and how the substance misuse services should support women and their babies throughout pregnancy and postnatally	Perinatal period	No
South Gloucestershire Safeguarding Children Board (2015)	Safeguarding Guidance for Substance Misuse [106]	England	Regional / South Gloucestershire/ Any setting	Social care teams and substance misuse providers	Provide clear understanding of practitioner’s role in assessing and supporting parents where their parenting has been identified as being impacted by drug use	Universal and applicable to all parents who use drugs but also has specific sections and references throughout relating to pregnancy, maternal and infant care	Perinatal period	Unclear
Rotherham Safeguarding Children Partnership (2016)	Safeguarding Unborn and Newborn Babies [107]	England	Regional/ Rotherham/Any setting	All staff working with children and families	The procedure is applicable to any practitioners who has identified a concern for an unborn baby and subsequently provides a framework for the appropriate safeguarding response and planning for practitioners, working together with families, to safeguard the baby at birth	The whole document is relevant as it covers the proceedings for pregnant women who have been referred to children's social care because their baby has been viewed as at risk, including for the use of significant substance use	Perinatal period	Yes
NHS England (2019)	Saving Babies Lives Care Bundle v.2 [108]	England	National / Any setting	Service providers, commissioners and healthcare professionals	Aim is to provide guidance on how to reduce infant mortality	There are a few specific recommendations in relation to PWWUD	Perinatal period	No
Shetland Child Protection Committee (2017)	Shetland Integrated Children's Services Plan [109]	Scotland	Regional/ Shetlands/ Any setting	All staff working with children and families	Set out strategic priorities, and aims to achieve the outcomes identified in the Shetland Partnership’s Local Outcomes Improvement Plan, Shetland’s Commission on Tackling Inequalities Report—On Da Level, and partner agencies Corporate Plans	The document highlights that PWWUD are included within the guidance as a group facing inequalities—therefore the guidance is relevant to them	Pregnancy	Yes
Southwark Safeguarding Children Partnership (2020)	Southwark Joint Service Protocol to meet the needs of children and unborn children whose parents or carers have substance misuse problems [110]	England	Regional / Southwark / Any setting	All staff working with children and families	Aims to ensure that professionals are aware of their safeguarding duties for children and support them to identify children who may be at risk as a consequence of parental substance misuse	The whole document is relevant specifically referring to pregnant women who use substances throughout	Perinatal period	Unclear
NHS Greater Glasgow & Clyde Alcohol and Drug Recovery Services Pharmacy Team (2019)	Standards for Drug & Alcohol Services in Community Pharmacies [111]	Scotland	Regional/ Scotland/ Community	Drug services & Community pharmacists	The guidance covers the prescribing and supervision of substitution therapy	The document contains references to the potential harms of detoxification during pregnancy and guidance related to opioid substitution therapies	Pregnancy	No
North Yorkshire Safeguarding Children Partnership (2019)	Substance Misuse in Parents [112]	England	Regional / North Yorkshire / Any setting	All staff working with children and families	Support local operational arrangements between adult and young people’s drug and alcohol service providers and children and families’ services, to ensure effective safeguarding and joint working	The whole document is relevant as applicable to parents who use drugs and specifically refers to pregnant women	Perinatal period	No
NHS Lothian—Anne Whittaker (2003)	Substance Use in Pregnancy [113]	Scotland	Regional/ Lothian/ Any setting	Professional and service providers working with women who use substances in pregnancy	To provide a framework and good practice guide for professionals supporting women who use drugs in pregnancy	The whole document is relevant to PWWUD	Perinatal period	Yes
Hull Safeguarding Children's Partnership (2022)	Substance Misuse in Pregnancy [114]	England	Regional / Hull / Any setting	All professionals working with parents expecting a child who misuse substances	To provide guidance to practitioners working with PWWUD	The whole document is specific to how people should work to support women using drugs during pregnancy and advice they should be given	Perinatal period	Unclear
University of Leicester Hospital Trust (2019)	Substance Misuse in Pregnancy – Guidance for the care of pregnant drug /alcohol users and their babies. [115]	England	Regional/ Leicester/ Hospital	Medical/Nursing staff	These guidelines are for community, maternity and neonatal staff to make sure that babies at risk of NAS are identified and receive proper care and management in the neonatal period	The whole document is relevant, as it outlines how midwifery staff should assess and support women who use drugs during pregnancy and babies at risk of neonatal abstinence syndrome	Perinatal period	No
Heart Of England NHS Trust (2016)	Substance Misuse in Pregnancy (V.4) [116]	England	Regional / Heart of England / Any setting	All staff working within maternity (Obstetrics, Midwifery, Anaesthetics, Neonatologists)	Purpose of document is to provide guidance on providing "excellent care" to PWWUD and their babies to reduce "maternal and neonatal morbidity and mortality associated with substance misuse in pregnancy."	The whole document is specific to this population	Perinatal period	No
Walsall Healthcare NHS (2017)	Substance misuse in pregnancy and subsequent care of the newborn [117]	England	Regional/ Walsall/ Hospital	Medical/Nursing staff	Provides written guidance for staff with regards to the correct procedures when caring for PWWUD. (inc. antenatal/postpartum)	The whole document is relevant	Perinatal period	Unclear
Royal Cornwall Hospitals NHS Trust (2020)	Substance Misuse in Pregnancy, Labour and Post Delivery Clinical Guideline [118]	England	Regional / Cornwall / Hospital	All healthcare practitioners supporting pregnant women who use substances	"This guideline aims to create an environment where women with problematic drug or alcohol use will have the knowledge of and confidence in a team who manage them sympathetically in pregnancy and help to minimise harm to the woman and baby. ^1^"	The whole document is specific to this population	Perinatal period	No
Frimley Health NHS Foundation Trust (2021)	Substance Misuse in Pregnancy: multidisciplinary guidelines for Frimley Health NHS Foundation Trust [119]	England	Regional/ Frimley/ Hospital	Medical/nursing staff	Outlines the clinical guidance related to women/families who use substance and their babies through partnership working with parents and multiagency collaboration	The whole document is relevant to PWWUD	Perinatal period	No
Welsh Government (2011)	Substance Misuse Treatment Framework (SMTF) Guidance for Evidence Based Community Prescribing in the Treatment of Substance Misuse [120]	Wales	National / Community	Professional prescribing drug treatment medications	Provides best available evidence to inform decisions about community prescribing treatment options for people who misuse substances	Has a specific section for substance misuse in pregnancy	Perinatal period	Yes
Birmingham Women and Children's NHS Foundation Trust (2021)	Substance Misuse: Management of Pregnant Women [121]	England	Regional/Birmingham/Hospital	Maternity/Neonatal staff	Gives guidance to nurses/midwives/doctors caring for PPWUD, are drug or alcohol dependent or in a drug treatment program	The whole document is relevant as it refers to the management of pregnant women who use drugs	Perinatal period	Yes
Public Health England (2013)	Supporting information for developing local joint protocols between drug and alcohol partnerships and children and family services [122]	England	National / Community	Service commissioners, providers and practitioners in drug and alcohol services, and children and families’ services	This document outlines what should be in local protocols for safeguarding children in families affected by drug or alcohol misuse	There is reference and recommended practice in relation to pregnancy and substance misuse	Perinatal period	No
Tayside Multi-agency Partnership (2021)	Tayside Multi-Agency Practitioner’s Guidance: Concern for Unborn Babies [123]	Scotland	Regional/Tayside/Any setting	Practitioners and service providers working with children	Provides practitioners and managers who might work directly or indirectly with children, young people and families with guidance on identifying and responding to concern about unborn babies	The generic guidance within the document is relevant to PWWUD as it highlights this group as a vulnerable population	Pregnancy	Yes
NHS Ayrshire & Arran (2019)	The Management of High-Risk Pregnancies [124]	Scotland	Regional / Ayrshire & Arran /Any setting	All staff working within agencies who are members of all three Child Protection Committees in Ayrshire	To provide clear guidance to staff managing high risk pregnancies	The whole document is relevant as it specifies women who use substances during pregnancy are high risk category	Perinatal period	Unclear
National Collaborating Centre for Mental Health (2018)	The perinatal mental health care pathway; Full implementation guidance [125]	England	National/Any setting	Mental health and social care providers	Introduces pathways outlining access to services for women with a mental health problem in the perinatal period, or with a history/existing mental health challenge who are planning a pregnancy	The document is universal guidance for perinatal mental health, and contains mention of the referral pathways from drug and alcohol services and contains recommendations for developing a care plan for this population	Perinatal Period	No
Norfolk & Waveney NHS Trust (2021)	Trust Guideline for the Care of Vulnerable Women in Pregnancy [126]	England	Regional / Norfolk /Any setting	Maternity services	To encourage the use of specialist services to support vulnerable women in pregnancy	The whole document is relevant as it specifies women who use substances during pregnancy are a vulnerable category, there are also specific recommendations for their care	Perinatal period	Yes
Hull Safeguarding Children's Partnership (2022)	Unborn Procedures and Guidance (Pre-Birth Pathway) [127]	England	Regional/Hull/Any setting	All staff working with children and families	The guidance has been designed by a multi-agency group, with the aim of developing a consistent Pre-Birth Assessment Pathway which identifies vulnerability early and provides a clear pathway into appropriate support services	The document is generic, but contains references to women who are pregnant and have substance misuse problems both within the guidance and in a dedicated section within the appendix	Perinatal period	Yes
Scottish Government (2015)	Universal Health Visiting Pathway in Scotland: Pre-birth to Pre-school [128]	Scotland	National / Community	Health visitors	Outlines the health visitors' role and schedule of care	Document is applicable as universal to all pregnant women, new mothers and babies. Recognises substance misuse by mother as category of vulnerability and makes a few specific recommendations for their care	Perinatal period	Yes
East Ayrshire Child Protection Committee (2017)	Vulnerable Pregnancy Procedure [129]	Scotland	Regional/East Ayrshire/Any setting	Healthcare professionals, especially those working with pregnant women and their babies	Aims to support practitioners conducting a needs led and person-centred assessment of vulnerable pregnant women, her partner and unborn babies that will identify strengths and risks. The assessment should be followed by a timely and proportionate response to any needs or risks identified	The whole document is relevant as problematic substance misuse—of either parent is listed as an identifying factor of a vulnerable pregnancy, however it does not contain specific recommendations for PWWUD	Perinatal period	Yes
West Yorkshire Consortium Inter Agency Safeguarding and Child Protection Procedures (2022)	West Yorkshire Consortium Inter Agency Safeguarding and Child Protection Procedure 1.4.15 Children of Drug Misusing Parents [130]	England	Regional / West Yorkshire /Any setting	All staff working with children and families	This is a child protection procedure for staff to follow in the assessment of safeguarding issues where a parent misuse substances	The procedure relates to all parents who use drugs including women who are pregnant or have just had a baby. There are also specific references to drug use in pregnancy and a section relating to this	Perinatal period	Unclear
Highland Council & NHS Highland (2019)	Women, Pregnancy and Substance Use: Good Practice Guidelines [131]	Scotland	Regional/Highlands/Any setting	Maternity services/Health and social care services	This guidance document represents best practice for maternity staff across Highland but notes that this is also applicable to other services involved with pregnant women who use substances	The whole document is relevant, as it outlines how midwifery services, and any other associated drug/alcohol services should care for PPWUD	Perinatal period	Yes
UK Government (2018)	Working Together to Safeguard Children A guide to inter-agency working to safeguard and promote the welfare of children [132]	England	National / Any setting	All staff and agencies working with children, and families; all local authorities, clinical commissioning groups, police and all other organisations and agencies	This document sets out legal child protection responsibilities of all agencies	The whole document is relevant as universal to all babies, and children. There are specific references to substance misuse in pregnancy, and more generally under the definition of neglect provided in the Appendix	Perinatal period	No
Derbyshire Safeguarding Children Board (2022)	Working with parents who are misusing substances [133]	England	Regional/ Derbyshire/ Any setting	All staff working with children and families	The protocol aims to ensure that all unborn babies have their needs identified and met as swiftly as possible to ensure that appropriate and timely services are delivered in an integrated manner	The document contains relevant sections on parental substance misuse and a Pre-birth Assessment Pathway for substance misuse	Perinatal period	No
Aberdeen City Child Protection Committee (2017)	Working with vulnerable unborn babies and their families multi-agency practice guidance [134]	Scotland	Regional / Aberdeen/ Any Setting	All staff working with children and families	Child protection guidance to support staff to be aware of impact of range of complex social circumstances (including substance misuse) on pregnancy and infants, identify unborn babies at risk, know their legal obligations and what action to take, and support them to share information appropriately	The whole document is specific to women who use drugs during pregnancy—as well as other identified factors that may place an unborn baby at risk of harm and require specific supports and interventions	Perinatal period	Unclear
World Health Organization (2014)	Guidelines for the identification and management of substance use and substance use disorders in pregnancy [135]	UK—wide	International/Any setting	All healthcare practitioners supporting pregnant/postnatal women who use substances	Provides evidence-based technical advice for healthcare providers regarding the management of substance use in pregnancy and facilitates healthcare practitioners’ application of "scientific principles of a public health approach" in their own national/local settings	The whole document is specific to women who use drugs during the perinatal period and postnatally	Perinatal period	Yes

**Table 4:** Best practice recommendations

Author (Year)	Document Title	Type of recommendations	Recommendations
Greater Glasgow and Clyde NHS (2016)	[CG] Use of alcohol and other drugs in pregnancy: guideline for management flowchart [26]	• Referral pathways • Practical clinical guidance / medical procedure • Prescribing protocols	• Provides flow charts for prescribing and management of pregnant women who are using illicit opioids, benzodiazepines, benzodiazepines and opioids together and who are already prescribed opioids • First visit; confirm pregnancy; take drug history, supervised urine dipstick; prescribing guidance = > diazepam 20mh/day • Opiate use; if prescribed, arrange of methadone prescription to be continued. If not prescribed, recommends methadone treatment at SNIPS (< 20mg/day, with daily review) • Benzodiazepine use; if prescribed and agrees to detox, refers to inpatient detox guidelines (external) If declines to detox, notify prescriber that detox has been recommended and declined; if not on prescribed benzodiazepines, offer inpatient detox • Opiate & Benzodiazepine use; recommends referral to SNIPs, potential commencement of methadone/community prescribing/in patient detox • In patient management of women already on substitute prescribing for opiate use; contact prescriber (SNIPs/non-SNIPs) to cancel script and commence inpatient prescribing • Women reporting illicit opiates necessitates immediate admission and stabilisation on prescribed methadone; transfer to SNIPS of existing maintenance methadone prescribing must be authorised by SNIPS; inpatient detox is advised for use of benzodiazepines; SNIPS staff should be notified to ongoing management plans
Greater Glasgow and Clyde NHS (2016)	[CG] Use of benzodiazepines in pregnancy. Guidelines for obstetric management [27]	• Philosophy of care / engagement • Referral pathways • Practical clinical guidance / medical procedures • Prescribing protocols	• Long term users of prescribed benzos should continue at the prescribed dose while in hospital; PWWU illicit benzodiazepines should undergo inpatient detox, and be transferred to SNIPS; PW on > 20mg prescribed benzodiazepines should also be transferred to SNIPS • Prescribing protocol for benzodiazepines detox (in-patient) in pregnancy: commence at maximum total daily dose of diazepam 30mg administered in 3 divided doses; reduce daily dose of diazepam by 5mg daily, reducing 3 doses in rotation with evening dose delivered last. Women to be accompanied / supervised at all times during inpatient detox • Uncontrolled use of illicit opiates = obstetric emergency; women should be immediately admitted and stabilised on methadone in accordance with SNIPS guidance; transferring to SNIPS maintenance methadone prescriptions should be authorised by SNIPS; inpatient detoxification is recommended for uncontrolled use of benzodiazepines • Problem drug use should be recorded in maternal case notes; inform women of health risks to baby • PWWUD should have abdominal circumference measurements (28–30 wks., and 32–34 wks.) and particular attention paid to abnormal parameters in CTGs
Greater Glasgow and Clyde NHS (2016)	[CG] Use of Opiates in pregnancy. Guidelines for obstetric management [28]	• Practical clinical guidance / medical procedures • Prescribing protocols • Child protection / safeguarding procedures	• Clinical guidance on substitute therapies, which includes recommending methadone/buprenorphine and highlighting that dihydrocodeine is not recommended; supervise OST, and adjust doses according to tolerance; detox is not recommended unless correct timing/high chance of success; • Recommendations for starting doses of methadone; starting dose of prescribed methadone = 5 – 20mg methadone in line with current level of use, and with escalation to the minimum dose that controls withdrawal symptoms; contains guidance for ongoing prescribing, noting not to prescribe above 40mg without consultation • Outpatient prescribing—SNIPS midwives can sanction 2 consecutive increments of methadone/buprenorphine doses, and reductions in methadone/buprenorphine doses can be authorised by SNIPS midwives on 2 occasions separated by a minimum of 24 h • Outpatient dispensing must always be in community pharmacies on a daily supervised basis; detailed guidance for inpatient dispensing • Overall guidance—record drug use; street opiate use is considered an obstetric emergency; community detox is preferred • Problem drug use should be recorded in maternal case notes; inform women of health risks to baby; PWWUD should have abdominal circumference measurements (28–30 wks., and 32–34 wks.) and particular attention paid to abnormal parameters in CTGs
BASW—Hulmes, A. and Galvani, S. (2019)	A child's first 1000 days: the impact of alcohol and other drugs [29]	• Overarching / organisational approach • Philosophy of care / engagement • Assessment	• Recommends non-judgemental approach; don't assume drug use constitutes automatic risk; be aware of father's substance use; do not overlook protective/resilience factors • Ask/record questions about parental substance use (don't assume others will) Holistic—biopsychosocial assessment
Blackpool Better Start. Centre for Early Child Development (2021)	A good practice guide to support the implementation of trauma informed care in the perinatal period [30]	• Overarching / organisational approach • Philosophy of care / engagement • Assessment	• Outlines a whole systems trauma informed care approach. Principles of trauma-informed care in the perinatal period are established as: Principle 1—Recognition and compassion Principle 2—Communication and collaboration Principle 3—Consistency and continuity Principle 4—Recognising diversity and facilitating recovery • Recommends the assessment of substance use, an understanding and supportive response to trauma disclosures, empowering women and respecting their choices • Clear referral pathways needed
Department of Health, Social Services and Public Safety NI (2012)	A Strategy for Maternity Care in Northern Ireland 2012–2018 [31]	• Overarching / organisational approach • Referral pathways	• Specifies 6 desired outcomes for maternity care: (list) These outcomes will be achieved via 22 objectives • Specific to PWWUD; Objective 9: there should be clear care pathways for women with long term health conditions who are planning a pregnancy and throughout their pregnancy • Contains clear schedule of care, and what should happen at each appointment • Advises recognition that disadvantaged women may be less likely to access maternity care before 12 weeks • Recommendation that GPs facilitate direct access to a midwife in the community
University Hospitals Birmingham NHS Foundation Trust (2019)	Abstinence Syndrome [32]	• Overarching / organisational approach • Assessment • Practical clinical guidance / medical procedures • Prescribing protocols	• Clinical guidelines on recognition/assessment of NAS: surveillance in hospital for four days post-natal (symptoms may be delayed); clinical guidelines for care of babies exhibiting symptoms of NAS; recommends women care for newborn baby as normal—skin to skin and breastfeeding encouraged apart from cases of high use of maternal benzodiazepine/crack/cocaine; includes discharge guidance • Check case conference decisions/discharge plans liaise with midwife • Pharmacological management of babies exhibiting NAS (inc. treatment with opioids/phenobarbital/chlorpromazine) • Provides chart to assess NAS score in babies
NHS Orkney & Orkney Island Council (2020)	Additional support pathway for women with vulnerabilities [33]	• Overarching / organisational approach • Referral pathways • Practical clinical guidance / medical procedures • Child protection / safeguarding procedures	• Recommends co-ordinated services—multiagency involvement & joint assessment; postnatal care discussed with multiagency team pre-discharge • Offer substance misuse intervention • Early consultation with specialist substance misuse midwife Provides flow chart for assessment of unborn baby at risk, which includes timescales and referrals • Provides timescales for child protection case conferences/pre-birth plans (case conference should take place before 28 weeks, or if late notification, within 21 working days of concerns being raised) • Notes importance of notification of appropriate health board if a women moves; protocol for Missing Family alert if concerns are raised about a child/family with no known address
NICE (2014; 2020)	Antenatal and postnatal mental health: clinical management and service guidance [34]	• Overarching / organisational approach • Assessment • Referral pathways • Practical clinical guidance / medical procedures • Prescribing protocols	• Clinical guidance on treatment options for women who use drugs • Referral to specialist substance misuse services • Multi-agency approach to be employed • Prescribing guidance related to benzodiazepines and detoxification, and treatment of babies post-birth • Mental health assessments should account for substance misuse; covers screening of babies post-birth
NICE (2021)	Antenatal Care [35]	• Overarching / organisational approach • Philosophy of care / engagement • Practical clinical guidance / medical procedures	• Conduct booking appointments as early as possible; offer additional or longer appointments if necessary • Advocates a sensitive, non-judgemental, and compassionate approach; personalise approach (tailor information to everyone) • Provide information to women about general health/wellness
Royal College of Obstetricians and Gynaecologists (2012)	Bacterial Sepsis Following Pregnancy. Green–top Guideline No. 64b, [36]	• Overarching / organisational approach • Referral pathways • Practical clinical guidance / medical procedures	• Guidance on medical management of injection site lesions and vascular access • Multiagency consultations recommended with local drugs advisory specialist team, neonatologists • Early referral to vascular access team
Lingford-Hughes, Welch, Peters and Nutt (2012)	BAP updated guidelines: evidence-based guidelines for the pharmacological management of substance abuse, harmful use, addiction and comorbidity: recommendations from BAP [37]	• Overarching /organisational approach • Philosophy of care / engagement • Referral pathways • Practical clinical guidance / medical procedures • Specific recommended interventions	• Multi-agency working (Information shared with GPs; provide access to integrated specialist care) • PWWUD should be fast-tracked into substance use treatment • Recommends psychosocial interventions for women using stimulants • Detox should be avoided in the first trimester (risk of miscarriage) • Offer personalised care
Bristol, North Somerset and South Gloucestershire Clinical Commissioning Group (2021)	Benzodiazepines and Z-drugs as Hypnotics and Anxiolytics [38]	• Practical clinical guidance / medical procedures	• Clinicians to be aware of the effects of benzodiazepines on neonates
Public Health England (2017)	Better care for people with co-occurring mental health and alcohol/drug use conditions [39]	• Overarching / organisation approach • Philosophy of care / engagement • Assessment • Referral pathways • Child protection / safeguarding procedures	• Assessment should be comprehensive and account for potential co-morbidities (PWWUD are likely to be experiencing co-occurring conditions) • Referral pathways into substance misuse/mental health programs/mental health • PWWUD are at risk of losing contact with services – recommends localised, innovative strategies and services models; services should foreground overcoming stigma, mistrust and barriers that may prevent access • Highlights the importance of the therapeutic alliance, adopting no-judgemental, empathic approach—responding to range of needs (holistic care) • Services should respond to a range of needs; multiagency working – collaboration with other services • Services should include safeguarding for children and vulnerable adults
Care Quality Commission (2018, reviewed 2019)	Brief guide: Substance misuse services – People in vulnerable circumstances [40]	• Overarching / organisational approach • Philosophy of care / engagement • Assessment • Practical clinical guidance / medical procedure	• Comprehensive assessment of needs, inc. factors such as mental health and housing (holistic) • Review, monitor and respond to changing needs • Daily records should detail treatment/recovery plans including actions regarding a client’s vulnerable circumstance; plan for emergency care if pregnant women are at high risk during opiate detoxification; monitor pregnancy and postnatal and offer postnatal support • Recommends multiagency working to meet the needs of the whole family
British Association for Psychopharmacology (2017)	British Association for Psychopharmacology consensus guidance on the use of psychotropic medication preconception, in pregnancy and postpartum 2017 [41]	• Overarching / organisational approach • Referral pathways • Practical clinical guidance / medical procedures • Prescribing protocols • Specific recommended interventions	• Recommends referral pathways to substance use services for women who use drugs • Prescribing guidance for methadone, buprenorphine (OST) for PWWUD; prescribing opioid maintenance treatment; pain management in labour; slow reductions in prescribed benzodiazepines • Facilitate early and effective antenatal care • Provision of integrated care which includes primary care, addiction services, obstetric and perinatal services • Information on Harm Reduction should be provided; provision of psychosocial interventions should be provided alongside pharma/medical care
Change, Grow, Live (2019)	Change, Grow, Live (CGL) Procedure: Substance Misuse in Pregnancy [42]	• Overarching / organisational approach • Philosophy of care / engagement • Practical clinical guidance / medical procedures • Prescribing protocols • Referral pathways • Specific recommended interventions	• Multi-disciplinary approach—Communication between multidisciplinary team is essential • Holistic needs assessment • Risk assessment and multi-agency planning meeting to be conducted before birth • Treatment/care goals should be realistic and tailored to woman • May have to split/ increase methadone dose in third trimester • Any reductions in medications should be gradual, and will need more frequent monitoring; stimulant use should be avoided, should consider risk and possible vaccinations for hep B & C and HIV • Should fast track pregnant service users to drug services • Offer psychosocial interventions (especially for drugs with no pharmacological interventions • Referral pathways flow chart with time scales is provided in Appendix
Aberdeen Alcohol & Drugs Partnership (2019)	Charter 3.2 Births affected by drugs (Health improvement plan) [43]	• Overarching / organisational approach • Philosophy of care / engagement • Practical clinical guidance/ medical procedures	• Multiagency collaboration; increase availability of harm reduction support • Advocates trauma informed approach and holistic assessment of needs • Support and review contraceptive offerings • Improve staff training and awareness; consider hidden populations and making 'every interaction count'
NICE (2017)	Child abuse and neglect [44]	• Specific recommended interventions	• Specific intervention models; 1) Parents Under Pressure 2) Additional home visiting programme
Outer Hebrides Drug and Alcohol Partnership and Outer Hebrides Child Protection Committee (2018)	Children affected by parental drug or alcohol related problems GIRFEC oriented inter-agency guidelines [45]	• Overarching / organisational approach • Referral pathways • Practical clinical guidance / medical procedures • Child protection / safeguarding procedures	• Midwife to refer on to other agencies (inc. social work) when pregnancy is confirmed; referral to senior staff if women repeatedly miss antenatal appointments • Decide on pre-birth assessment at 28 weeks and discharge plan • Multiagency referral and assessment Flow chart provided for assessment timescale and referral pathways • NAS necessitates automatic referral to social worker—mother & baby undergo 5-day assessment in hospital
HIPS Safeguarding Children Partnership (2022)	Children living in households where there is substance misuse [46]	• Overarching / organisational approach • Referral pathways • Child protection / safeguarding procedures • Specific recommended interventions	• Women using substances will be identified and referred to substance misuse team Holistic assessment and family-based approach to be used • Highlights specific approach to care planning; The Care Planning Approach / Care Co-ordination Approach which includes input from the link midwives and a social worker from Children's social care, who will be invited to any meetings taking place in respect of the child/ren • If a new-born requires treatment to withdraw from substances, an assessment and a pre-discharge discussion should take place and considerations regarding making a referral to Children's social care in line with the Referrals Procedure before discharge should be considered • The needs of child should take precedent over confidentiality concerns of substance use services
Hull Safeguarding Children Partnership (2022)	Children of parents or carers who misuse substances [47]	• Referral pathways • Child protection / safeguarding procedures	• Where a practitioner is working with a pregnant woman who is using substances and has concerns that their parenting capacity may be compromised, make appropriate child protection referrals • Highlights risks and potential harms to children • Suggests a family-based approach is adopted • New-borns with NAS should be referred to child protection services
Regional Child Protection Procedures for West Midlands (2022)	Children of parents who misuse substances [48]	• Philosophy of care / engagement • Referral pathways	• Provides clear referral pathways to social work services and substance misuse team • Recommends use of a Care Planning / Care Co-ordination Approach which involves making sure that all professionals involved are invited to key meetings etc • Stresses that the father should be identified and involved in any assessment
South Lanarkshire Partnership (2021)	Children's Service Plan: 2021–2023 [49]	• Overarching / organisational approach • Philosophy of care / engagement	• Recommends developing and supporting services which focus on prevention and early interventions for parents using substances during pregnancy • Recommends a trauma-informed, children’s rights-based approach overall
HM Prison Service (2000)	Clinical services for substance users [50]	• Overarching / organisational approach • Referral pathways • Prescribing protocols	• Referral to NHS consultant obstetrician; involvement of specialist health staff with prison health care staff • Multiagency guidelines for PWWUD, in conjunction with obstetrician and NHS substance use specialist • Refers to evidence-based guidelines for prescribing for PWWUD
NICE (2016)	Coexisting severe mental illness and substance misuse: community health and social care services [51]	• Overarching / organisational approach • Philosophy of care / engagement • Assessment • Referral pathway • Child protection / safeguarding procedures	• Staff in all organisations should consider the varied needs of the population, including physical health problems, homelessness or unstable housing • Practitioners should be mindful that this population may not access services in a timely manner because of stigma, feelings of coercion, mistrust of services • Referrals should be made onto substance use, or relevant support services, as unmet need may trigger a relapse. It suggests practitioners provide direct help or refer onto specialist agencies. Practitioners should ensure a woman’s safeguarding needs are met • Outlines referrals to mental health services, assessment and care planning. This should be person-centred and involve the person’s family and carers • Trauma informed approach recommended • A multi-agency approach/partnership working is recommended to address physical health, social care, housing, pregnancy, childcare and other support needs • Services should be made more inclusive, accessible and more user-friendly for people who use drugs/have co-existing mental health conditions; recommends offering face-to-face and telephone appointments, outlines recommendations for maintaining contact with patients
Derbyshire Safeguarding Children Board (2020)	Derby and Derbyshire Multi Agency Protocol for Pre-Birth Assessments and Interventions [52]	• Overarching / organisational approach • Philosophy of care / engagement • Assessment • Referral pathways • Practical clinical guidance / medical procedures • Child protection / safeguarding procedures	• Conduct routine assessments and recording of drug use; ask about partner's/significant adult's drug use • Refer to substance use services if necessary; refer complex/concerning cases to child protective services • Ensure drug services support is in place > 24 weeks; recommends multi-agency working • Practitioners should consider concurrent vulnerabilities (domestic abuse, housing, dual diagnoses) (holistic assessment) • Includes flow chart with specific timescales for child protection assessment stages and pre-birth conference to be held at 28 weeks
The Royal College of Psychiatrists and the Faculty of Forensic and Legal Medicine (2020)	Detainees with substance use disorders in police custody: Guidelines for clinical management [53]	• Overarching / organisational approach • Referral pathways • Practical clinical guidance / medical procedures	• Practical clinical guidance on the procedures for the assessment, treatment, and referrals to be made for women held in police custody who are pregnant and have issues with substance misuse; includes pregnancy testing, examination, and prescribing
Department of Health -Clinical Guidelines on Drug Misuse and Dependence Update 2017 Independent Expert Working Group (2017)	Drug misuse and dependence UK guidelines on clinical management [54]	• Philosophy of care / engagement • Practical clinical guidance / medical procedures • Prescribing protocols	• Clinical guidance document that provides clinicians with clear recommendations as to the approach, and procedures to be followed when treating women who use drugs throughout the perinatal period. • Advises trauma-informed care approach • Advises early assessment of risk and needs with a case conference for unborn babies if at risk of harm, should include parents, and follow integrated care pathways. (Holistic assessment) • Multi-agency assessment, and clear joint working protocols between domestic abuse and drug services. PWWUD in prison to receive care from multi-disciplinary team • Advice on stabilising mother in 1st trimester and detoxification in 2nd trimester and specific treatment recommendations • Suggests balance in reducing drugs, in terms of risk of withdrawal vs risk of patient increasing use • Encourages breastfeeding
Doncaster and Bassetlaw Teaching Hospitals NHS Foundation Trust (2017)	Drug Misuse Management in the Acute Hospital Setting – guidelines [55]	• Overarching / organisational approach • Referral pathways • Prescribing protocols • Specific recommended interventions	• Encourage a sensitive approach when working with PWWUD; attempt to integrate into mainstream services • Guidance for OST prescribing in pregnancy; warns against sudden withdrawal of opioids; guidance on initiating methadone prescribing • Harm reduction re: avoidance of withdrawal • Urgent referral pathways to integrated care teams, including specialist midwifes, obstetricians and Drug and Alcohol liaison nurse specialist)
National Collaborating Centre for Mental Health (2019)	Drug misuse: Opioid detoxification The NICE Guideline [56]	• Assessment • Practical clinical guidance / medical procedure	• Assessment—needs to be comprehensive and include drug using history, any other social issues, mental health problems, risk behaviours (holistic) • For PWWUD, detoxification should only be undertaken with caution • An examination of patient's physical and psychiatric health is important to assist in the diagnosis of dependence and to assess any further complications to the detoxing process (such as pregnancy); consider the patient's social and personal circumstances, including finances, housing, social support, criminal status etc • Co-morbid physical or mental health problems should be treated in conjunction with opioid dependence
NHS (2021)	Equity and equality Guidance for local maternity systems [57]	• Philosophy of care / engagement • Practical clinical guidance / medical procedures	• Ask women with complex social factors about acceptability of services • Co-produce services according to local need • Provides a good practice example where a trauma—informed care approach was adopted • Record the number of women who complex social factors and presentation time
NHS Lothian (2021)	Expressed breast milk: Information for carers of vulnerable babies [58]	• Practical clinical guidance / medical procedures	• Women to be encouraged to breast feed, to develop attachment between infant and mother, except for those using cocaine
Scottish Government (2021)	Families Affected by Drug and Alcohol Use in Scotland: A Framework for Holistic Whole Family Approaches and Family Inclusive Practice [59]	• Philosophy of care / engagement • Specific recommended interventions	• Key messages about adopting a holistic, trauma informed, and family approach. This involves offering practical support to address wider issues such as poverty and housing as well as ensuring that the whole family is included • Recommends the "Safe & Together" domestic abuse intervention model
North Lanarkshire CPC & South Lanarkshire CPC & Lanarkshire ADP (2015)	Getting it Right for Children and Families Affected by Parental Alcohol and Drug Use in Lanarkshire [60]	• Overarching / organisational approach • Assessment • Referral pathways	• Holistic approach to assessment • Continuous risk assessment throughout pregnancy • Referral pathways to good antenatal care, and to specialist midwife (inc. Lanarkshire Additional Midwifery Service—LAMS) Integrated and multi-agency working
(ELBEG-PP 2013) Whittaker, A., Templeton, L., Mitchell, F., Hill, L. & Neilson, A. (2013)	Getting it right for children and families affected by parental problem alcohol and drug use: Guidelines for agencies in Edinburgh and the Lothians. [61]	• Overarching / organisational approach • Philosophy of care / engagement • Assessment • Practical clinical guidelines / medical procedures	• Co-ordinated Multi-Agency Working/Whole-family Approach/Holistic approach; professionals and agencies working together to create a comprehensive packaged of care during the antenatal/postnatal period • Encouraging breastfeeding in women who use drugs, unless the woman is HIV positive • Continuous Risk Assessment throughout care; and Integrated Assessment; this approach ensures all practitioners and agencies involved with the family are invited to contribute to the integrated assessment, including a multi-agency meeting organised for prior to 24 weeks gestation • Engage with fathers-to-be and involve them in all aspects of the care process
Scottish Government (2013)	Getting Our Priorities Right (GOPR) [62]	• Overarching / organisation approach • Philosophy of care / engagement • Practical clinical guidance / medical procedures • Child protection / safeguarding procedures	• Multiagency working; follow the Universal Care Pathway; multidisciplinary assessment and prepare an inter-agency plan prior to birth • Part of multiagency assessment involves potential contact with a social worker - • Provide effective antenatal care—Health visitor to conduct a full GIRFEC assessment (may take up to 6 months) and allocate a core or additional health plan indicator • Babies should remain with their parents where possible Parental drug use does not necessarily mean children will be adversely affected; any statutory involvement must be justified; substance misuse should prompt assessment, and this should be holistic—including wider social context and need
Forth Valley Drug Partnership and Child Protection Committee (2019)	Getting Our Priorities Right for Children and Families affected by Parental Alcohol and Drug Use: Guidance from the Forth Valley Alcohol and Drug Partnerships and Child Protection Committees [63]	• Philosophy of care / engagement • Referral pathways • Child protection / safeguarding procedures	• Women should be encouraged to disclose substance misuse and that worker should have non-judgemental approach to encourage engagement with antenatal and drug services • Provides guidance on referrals to be made to specialist services – pre-birth planning service, drug, and alcohol services • Highlights the potential need for child protection procedures—pre-birth child protection conference (to be conducted ASAP)/ post birth child protection case conference, unborn / new-born babies being placed on the child protection case register, and the importance of information sharing • Holistic and trauma informed care approach to be adopted
East Ayrshire Child Protection Meeting (2014)	Good Practice—Working with Pregnant Women with Parental Substance Misuse [64]	• Philosophy of care / engagement • Assessment •Child protection / safeguarding procedures • Specific recommended interventions	• Key messages about approach and engagement tools. e.g., Effective communication, named support worker (i.e., continuity of care), motivational interviewing • Recommends a universal model of care and clear care pathways—Flow chart provided in the Appendix • Child protection referral to be made to SW, and initial CP case conference to be held before 28wks, or within 21 days if late pregnancy. Pre-birth assessment forms and tools in Appendix • Case manager with empathic relationship with parent (again continuity of care); lead professional and multiagency meetings and assessment; postnatal support from the same worker (continuity of care) • Intensive parenting programmes recommended to improve outcomes
Department of Health, Social Services, and Public Safety (2020)	Guidance for Alcohol and Drug services in Northern Ireland to best deliver treatment and care during the COVID-19 pandemic [65]	• Prescribing protocols	• OST prescribing guidance during Covid-19; recommends maintaining access to OST and injection equipment must be a high priority • States that Buprenorphine is preferred to methadone with the caveat—except perhaps in pregnancy
NHS Grampian (2019)	Guidance for the use of buprenorphine products for the treatment of opioid dependence in NHS Grampian [66]	• Philosophy of care / engagement • Assessment • Practical clinical guidance / medical procedures • Prescribing protocols	• Pregnant women can remain on methadone/buprenorphine, if informed of risks; transferring to buprenorphine is not advisable during pregnancy because of risk of precipitated withdrawal (and potentially of inducing withdrawal of the foetus). However, refer to evidence that buprenorphine may result in lower NAS severity • Consider the impact of buprenorphine on labour pain management plans (as they may impact the management of acute pain) (ES) • Risk–benefit assessment should be conducted on mothers stabilised on buprenorphine who wish to breastfeed •General patient best-practice to encourage breastfeeding • Provide a balanced view of each OST medication to allow patients to make an informed decision; treatment should be tailored to the individual and combined with psychological/social interventions for greater chance of success; opioid dependence should be diagnosed prior to treatment commencing
Barnsley Safeguarding Children Partnership (2022)	Guidelines for multi-agency assessment of pregnant women and their babies in cases where there is substance misuse [67]	• Overarching / organisation approach • Philosophy of care / engagement • Referral pathways • Child protection / safeguarding procedures	• Substance use disclosure necessitates referral to Hospital's Specialist Drug and Alcohol Midwife; fast-track women into substance recovery services • Encourage partner to access substance recovery services; conduct early assessments where possible • Provide sensitive and non-judgemental care/information • Outlines child protection proceedings and for potential pre-birth child protection conferences which should take place at 28 wks • Recommends multiagency assessment and working, and coordinated clear birth-plans • It should be explained to women that they should expect to remain in hospital for 5–7 days for the baby to monitored / treated for NAS
Leeds City Council (2010)	Guidelines for the assessment of parental substance misuse [68]	• Referral pathways • Child protection / safeguarding procedures	• A full child and family assessment to be conducted—covering three main categories: parenting capacity; child's developmental needs; environmental factors. Effort to be made to validate information provided •Workers should not assume that substance use necessarily mean there are child protection concerns, but if there are concerns of risk of harm a referral to be made to social work children’s team
St Mungo's (2017)	Homeless Pregnancy Toolkit [69]	• Philosophy of care / engagement • Assessment • Referral pathways	• Stresses the need for multiagency working; suggests a lead professional can help with engagement and coordinating multiagency work to identify risk. Risk assessment to also consider their partner • Neutral, non-judgemental advice and support is integral to supporting homeless pregnant women who may have complex trauma and complex social needs, and maybe uncertain about their pregnancy. (trauma informed and holistic) • Referrals and information sharing need to be timely as women’s circumstances and living circumstances can change quickly and frequently • Recommends referrals to specialist midwifery service, and drug / alcohol services •Flow chart with timeline of schedule of care and referrals to be made provided
NHS (2017)	Implementing Better Births, a resource pack for local maternity systems [70]	• Overarching / organisational approach • Philosophy of care / engagement • Assessment • Specific recommended interventions	• Stresses continuity of care (women to have the same allocated worker throughout), clear referral pathways, and women to be given choices about their birth and care • Recommends every pregnant woman should have a personalised care plan, created in collaboration with the woman, respecting their rights and choices—provides guidance on how to have a supportive dialogue to facilitate this, and conduct a holistic assessment of need • Recommends local services building services around a community hub model
Glasgow Child Protection Committee (2008)	Inter-Agency procedural guidance for vulnerable women during pregnancy [71]	• Overarching / organisational approach • Philosophy of care / engagement • Assessment • Referral pathways • Child protection / safeguarding procedures	• Babies should remain with their parents as long as possible • Confidentiality and information sharing procedures should be transparent and clear • Child protection concerns should be addressed in a Pre-Birth conference held between 28–32 weeks • Parents (inc. fathers) should be involved in addictions treatment and support; women should be referred to Women's Reproductive Health Services for specialist care • Interagency working should include Post-birth plans, and procedures for immediate child protection proceedings; Hospital-based antenatal clinics/inpatient wards should alert hospital based social work units of any safeguarding concerns that arise • Assessment is an ongoing, collaborative process between agencies and parents; clear, effective communication is essential; parents should be kept informed or Interagency Child Protection Procedures, and the purpose of discussions/conferences. Should be holistic •Flow chart of clear referral pathways, and Special needs in Pregnancy pathways are provided
NICE (2014; 2017)	Intrapartum care for healthy women and babies [72]	• Assessment	• Pregnant women who use drugs recreationally should have individual assessment when planning place of birth
NICE (2019)	Intrapartum care for women with existing medical conditions or obstetric complications and their babies [9]	• Assessment	• Women who use drugs should have an individual assessment when creating a birth plan and planning for the place of birth
Wilson, C., Boxhall, C. and Kelleher, M. (2019)	Lambeth drug and alcohol service guidelines for the management of substance misuse in the perinatal period [73]	• Philosophy of care / engagement • Assessment • Referral pathways	• Comprehensive assessment to be conducted •Routine enquiry into substance misuse (inc. prescribed medication) • Routine enquiry should be conducted sensitively • Any pregnant women presenting to addictions must be referred to the midwifery team immediately
Royal College of Obstetrics and Gynaecology (2010; 2017)	Late intrauterine foetal death and stillbirth [74]	• Practical clinical guidance / medical procedures	• Recommends testing maternal urine for potential hidden drug use (cocaine), with mother's permission
NICE (2021)	Looked-after children and young people [75]	• Overarching / organisational approach • Philosophy of care / engagement • Child protection / safeguarding procedures	• Consider professional support for birth parents with substance misuse challenges, that can help with reunification • Recommends the provision of ‘relational, emotional and mental health support’ alongside court/child protection proceedings, and to continue mental health and drug abstinence support after proceedings/reunification Encourages trauma-informed care, and that trauma-informed training should be integrated into existing training offerings
University Hospitals Birmingham NHS Foundation Trust (2020)	Management of substance misuse in pregnancy [76]	• Overarching / organisational approach • Philosophy of care / engagement • Referral Pathways • Practical clinical guidance / medical procedures	• PWWUD to be looked after in Substance Misuse Antenatal Clinics (multidisciplinary team); Referred to drugs services • Maternity staff should be aware of signs/risk of domestic abuse in pregnancy; follow-up women who do not attend routine appointments; outlines drug-specific recommendations • Ensure multiagency discharge planning and pre-birth conferences are organised as necessary • Record drug use in a respectful, confidential and accurate way • Includes referral pathway/assessment flowcharts and week to week care schedules • PWWUD are advised to stay in the hospital for a minimum of 72 h so that any symptoms of NAS can be managed
University Hospitals Plymouth (2019)	Management of substance use during pregnancy and the postnatal period [77]	• Referral Pathways • Practical clinical guidance / medical procedures • Child protection / safeguarding procedures	• Risk assessment to be conducted for other children involved to assess potential safeguarding concerns • Referral pathways to specialist midwifery team and drug services • Breast feeding benefits generally outweigh risks, and women should be able to make an informed decision • Babies should be observed for NAS for a period of up to 5 days (120 h), dependent on NAS scoring
Royal College of Obstetricians and Gynaecologists (2011)	Management of women with mental health issues during pregnancy and the postnatal period [78]	• Philosophy of care / engagement • Assessment • Referral pathways	• Antenatal booking visit -Women to be asked sensitively about any history of using illegal drugs, along with other social / contextual factors such as domestic abuse, previous trauma, social supports etc., (Holistic assessment) • Women with alcohol or drug misuse should be referred to addiction services
NHS York Teaching Hospital (2019)	Maternity services guideline: Antenatal appointments guideline [79]	• Philosophy of care / engagement • Assessment • Referral Pathways	• At antenatal booking appointment, ask sensitively about history of drug use (alongside questions about IPV, sexual abuse or assault, mental health and social support), as this group is especially vulnerable to depression/suicide in pregnancy • PWWUD should be referred to addiction services in line with local protocol • Aims to provide evidence-based, holistic recommendations for care of PWWUD • Contains detailed, week-by-week schedule of care and referral pathways flowchart
Evelina London and NHS Guy's and St Thomas' NHS Foundation Trust (2021)	Maternity substance misuse in pregnancy guideline [80]	• Philosophy of care / engagement • Referral pathways • Practical clinical guidance / medical procedures • Child protection / safeguarding procedures	• Recommends that maternity services adopt a sensitive approach pregnant women who use drugs – that they “feel listened to, and their opinions respected”. Maternity care to focus on pregnancy rather than drug use. Birth plan to reflect women’s choices • Threshold for child protection referral where women use substances is low • Clinical practice recommendations around prescribing pain relief in labour, and urine testing. Breastfeeding to be encouraged • Includes flowchart of referrals and care pathways • Does not specify time a women must remain in hospital but notes that a midwife should begin withdrawal observations on the neonate after birth and continue for up to five days
Wolverhampton Safeguarding Children Board (2013)	Multi-Agency Guidance Hidden Harm – Parental substance misuse and the effects on children [81]	• Overarching / organisational approach • Philosophy of care / engagement • Assessment • Referral pathways	• Parents who use drugs can be 'good enough' parents, (not necessarily neglectful) • Effectively share information (multiagency sharing); conduct ongoing assessments • Follow outlined care pathways and referrals • When addressing families where substance use is present, consider other children, and make sure assessment is holistic
Scottish Government (2021)	National Guidance for Child Protection in Scotland 2021 [82]	• Philosophy of care / engagement • Assessment • Referral pathways • Child protection / safeguarding procedures	• Child protection guidance which sets out that all agencies have a responsibility to recognise risks to the child Included within the definition of neglect provided is harm to unborn babies through drug or alcohol use • Recommends GP and hospitals must be mindful of domestic abuse especially in specific circumstances—women who are pregnant and have drug and alcohol difficulties • Community pharmacists to monitor children of parents who use drugs and addiction services • Prebirth assessment and support suggests where drug use is one alongside other risk factors e.g., previous child removal an Inter-agency referral discussion (IRD) should be triggered. Pre-birth assessment should begin asap where there is risk of significant harm. There should be multi-agency working, a clear plan for the child once it is born, and strengths should also be recognised • Pre-birth case conferences (called Child Protection Planning Meeting’s) are to be held within 28 calendar days of the concern being raised and within 28 weeks of gestation • Families, and children who may be removed, deserve trauma informed care to support them and minimise harm • Care and assessment should also be person-centred and holistic • Includes child protection referral pathways
Highland Council & NHS Highland (2020)	North Highland Vulnerable Pregnancy Pathway—Taking a trauma informed approach in understanding and responding to vulnerability in pregnancy [83]	• Overarching / organisational approach • Philosophy of care / engagement • Assessment • Referral Pathways • Child protection / safeguarding procedures	• Outlines week-by-week antenatal and child safeguarding procedures for vulnerable women, including initiation of case conferences, Health Plan Indicators and continuous assessment • Recommends SHANARI Wellbeing Assessment tool, multiagency working and that information should be collated by agencies into a single agency chronology. If necessary, a multi-agency chronology can be compiled by the Lead Professional • Advocates for building trusting relationships based on choice and collaboration empowering families through a trauma informed approach • Includes a schedule of care; Pregnancy Pathway
Staffordshire, Shropshire & Black Country Newborn and Maternity Network and Southern West Midlands Maternity and Newborn Network (2017)	Obstetric Guidelines 2017–19 [84]	• Child protection / safeguarding procedures • Referral pathways • Practical clinical guidance / medical procedures • Prescribing protocols	• Book consultant care and refer to specialist midwife • Explicit permission is required to record substance use in handheld notes, as these are readily available (cannot guarantee privacy) • Advise Hep C screening, alongside routine HIV and Hep B screening; women who are not booked should be screened for blood-borne viruses • Women are encouraged to begin opioid maintenance programs; consider increasing doses in 3rd trimester to avoid sudden withdrawal; maintain contact with specialist drug worker, and encourage attendance if non-attendance • In labour: prescribe usual methadone dose and inform anaesthetist and neonatologist • Postpartum: Encourage breastfeeding, maintain multidisciplinary working; Multiagency discharge planning should be in place, including a referral to children’s services if necessary
NICE (2022)	Opioid dependence: Scenario: Managing special circumstances [85]	• Philosophy of care / engagement • Referral pathways • Practical clinical guidance / medical procedures • Child protection / safeguarding procedures	• Key messages about how workers should approach and engage with pregnant women and mothers who use drugs: women may be afraid of being judged, and social services. Treat them same way as all other pregnant women. Emphasise importance of attending healthcare appointments. Try to involve women's partner if appropriate • Refer to social services if needed and offer referral to drug treatment services • Provides details of recommended practice for prescribing drug treatments during pregnancy • Recommends breast feeding except if using high-dose benzodiazepines, cocaine/crack, or HIV positive
Orkney Health and Care (2021)	Orkney Alcohol and Drugs Partnership Strategy 2021–31 [86]	• Overarching / organisational approach • Philosophy of care / engagement • Referral pathways	• An overarching local drugs strategy that has key messages about a trauma informed, holistic and family centred approach. Stresses early identification, intervention, multi-agency working and information sharing. Recommends an overall recovery focused model of care • The strategy recognises women use drugs and are pregnant have specific needs and need access to alcohol and drug treatment during pregnancy and after childbirth
Outer Hebrides Community Planning Partnership (2020)	Outer Hebrides integrated children's services plan [87]	• Overarching / organisational approach • Assessment • Referral pathways	• Create an embedded system in maternity services to regularly review and assess vulnerable parents to arrange appropriate targeted support as necessary (continuous assessment) • Implement multi-agency working sooner in the care process; create effective pathways for vulnerable groups (such as referrals to Vulnerable in Pregnancy), who can provide specialist care Ensure all support and assistance provided to families is trauma-informed and holistic, addressing issues such as mental health and poverty
The Royal College of Midwives (2020)	Parental emotional wellbeing and infant development [88]	• Overarching / organisational approach • Philosophy of care / engagement • Referral pathways	• Ask about substance use sensitively, without a partner present; be available to provide support (talking, listening, understanding) • Flexible appointments; ensure confidentiality where possible; develop clear multi-agency protocols and referral pathways (social care/third sector) • Involve referrals to social services for pre-birth assessments/interventions as necessary • Recognises that for women who have experienced trauma, birth can be a challenging time, and can exacerbate existing trauma (doesn't mention trauma-informed care explicitly)
Milton Keynes Inter-Agency Safeguarding Children Procedures (2022)	Parental Substance Misuse [89]	• Assessment • Referral pathways • Child protection / safeguarding procedures	• Stresses that agencies should work in partnership, and the need for information sharing between substance misuse workers, maternity services and social workers. All agencies to contribute to case discussions, pre-birth and child protection case conferences • If a woman is pregnant and using drugs, early assessment to be made. If there are concerns, she or her partner is "significantly" using drugs then a referral to be made to children's services (CS). If baby is born with NAS immediate referral to CS to be made • If there has been a previous child taken into care, the woman has been using heroin, methadone, cocaine or comparable substances for a significant period; or is continuing to use heroin or misuse methadone and not preparing for her baby’s arrival a referral must be made to CS
Public Health England (2021)	Parents with alcohol and drug problems: adult treatment and children and family services [90]	• Overarching / organisation approach • Assessment • Referral pathways • Child protection / safeguarding procedures	• Staff should be trained in asking families about alcohol/drug use; staff training should encompass “the skills and confidence of a wide range of professionals” including schools, mental health, criminal justice and care settings to identify potential areas families may need support in • Recommends a trauma-informed approach, which can improve engagement in services • Data should be collected and collated on prevalence of families affected by drug/alcohol use in the area; information sharing agreement between adult and children’s services will help with the identification of need early and ensure initial and continual assessment is carried out • Recommends collaborative assessment; senior leaders should develop a partnership/multiagency system with links to child services; Continuous assessment—services should regularly monitor parental substance use and parental arrangements for potential safeguarding issues; A substance misuse lead in each service can act as a main point of contact and proceed with referrals to drug treatment services • Referrals from children and family services into drug/alcohol treatment should be considered high priority referrals; drug treatment services can also consider the wider social needs of the family and make referrals to threshold support services (family hubs etc.)
Scottish Government (2020)	Perinatal & Infant Mental Health Programme Board 2020–2021 Delivery Plan [91]	• Overarching / organisational approach • Philosophy of care / engagement • Practical clinical guidance / medical procedures • Specific recommended interventions	• Collaborative and joint working between agencies is essential to ensure continuity of care • Develop peer support and family support (inc. partners/kinship carers), and that family support services be holistic • Establish a working group for greater analysis and dissemination • Increase staffing levels and provision across maternity and antenatal services, including specialist midwives and psychological services. Increase digital access to services Develop initiatives and resources for workers in specialist PNMH services. Increase capacity by rolling out training programme • Recommends peer support intervention programme
Sussex Partnership NHS Foundation Trust (2018)	Perinatal Mental Health: Prescribing guidance for trust prescribers and GPs [92]	• Referral pathways	• Women who use drugs who are pregnant should be referred to local substance use services to receive specialist care with input from neonatology and obstetrics
NICE (2021)	Postnatal Care [93]	• Overarching / organisational approach • Referral pathways	• PWWUD should be referred to local substance misuse • Management of PWWUD should involve multi-agency collaboration (lead by a substance misuse specialist, ideally including input from neonatology and obstetrics)
Dumfries and Galloway—– Strategic Pre-Birth Planning Group (2019)	Pre-birth assessment protocol for vulnerable pregnancies [94]	• Philosophy of care / engagement • Assessment • Referral pathways • Child protection / safeguarding procedures	• Overall, recommends that professionals undertake holistic, needs led/person centred assessments for vulnerable pregnant women and unborn babies • Information sharing is best practice, but this is overridden by child safety • If vulnerabilities are identified, midwife to liaise with specialist pre-birth team; all vulnerability team enquiries to be screened by social worker and specialist midwife • Accurate chronology to be taken from notification of pregnancy • Pre-birth assessment to be undertaken where vulnerabilities identified; If identified as necessary by the Pre-Birth Assessment, Initial Child Protection Case Conference meeting will be held at 28 weeks to formulate a plan for the child; additional reviews may be conducted if the baby presents with NAS; if a baby presents with NAS without prior notification of substance use, Social Work to convene and Initial Referral Discussion, (to be held same day) • Babies exposed to maternal substance use or prescribed substitution therapy are required to stay a minimum of 72 h, for observation • Face-to-face handover to health visitor; Lead professional is always a social worker who coordinates multi-agency assessment, if problematic substance use has been identified • Contains pre-birth process map/flowchart outlining procedure and referrals
NICE (2010; 2018)	Pregnancy and complex social factors: a model for service provision for pregnant women with complex social factors [95]	• Overarching organisational approach • Philosophy of care / engagement • Referral pathways	• PWWUD need supportive and coordinated care during pregnancy • To address barriers PWWUD face accessing services attention is paid to: integrating care from different services; making sure staff attitudes do not prevent women from using services; tackling women's fears about the involvement of children's services and possibility of their baby being taken into care, providing information specific to their needs; supporting women to address feelings of guilt about their misuse of substances and possible effects on their baby • Recommends co-ordinated antenatal care across services; one single care shared plan. PWWUD should be allocated a named specialist midwife or doctor who is accessible to them • Healthcare staff to receive training on needs of PWWUD, and reception staff etc. trained on how to respond sensitively • PWWUD should be referred to substance misuse service. A variety of engagement & communication methods used. Information to be given on services available, potential harms to baby, and transport options for attending appointments
NHS Lothian (2011)	Pregnancy and problem substance use [96]	• Philosophy of care / engagement • Assessment • Referral pathways • Practical clinical guidance / medical procedures • Child protection / safeguarding procedures	• Recommends a non-judgemental approach, holistic assessment, multiagency working, and involvement in planning during pregnancy and once the baby is born. Family support plan in place before baby born. Including fathers is vital • Drug treatment – women should not feel pressured into stopping drugs. Discuss treatment options with parents and recognise pregnancy as a time mothers and fathers are receptive to harm reduction and improving their health • Promote breastfeeding unless the woman is HIV positive • Child Protection – drug use doesn’t necessarily mean risk of harm to baby / infant – if there is concern child protection procedure to be followed • Postnatal, keep mother and baby on ward for 72 h to monitor for NAS. A strengths-based approach which aims to enhance parenting capacity and interventions which target couples and families rather than parents as individuals
NHS Lothian Quality Prescribing Group Substance Misuse Directorate (2016)	Pregnancy Guidance [97]	• Overarching / organisational approach • Practical clinical guidance / medical procedures • Referral pathways • Prescribing protocols	• Refer women in Edinburgh with additional needs to PrePare • Maintain good communication between agencies • Opioid users should begin pharmacotherapy, alongside psychosocial interventions; women using cocaine/stimulants should be advised to stop and offered psychological therapy/family intervention; women on benzodiazepines should be stabilised on diazepam. Women should be maintained on a dose that stops or decreases illicit drug use • Take into consideration pain management plan in labour, including potentially low threshold for epidural; recommends access to skilled paediatric care • Continue support (advice/treatment) postnatally; encourage breastfeeding, except in cases of crack/cocaine/high benzodiazepine use
Ministry of Justice and HM Prison and Probation Service (2021)	Pregnancy, mother and baby units (MBUs), and maternal separation from children up to the age of two in women’s prisons [98]	• Overarching / organisational approach • Philosophy of care / engagement • Assessment • Practical clinical guidance / medical procedures	• Operational policy for mother and baby units in prisons includes key messages around information sharing, and multi-agency case management • Prisons must consult healthcare on the appropriate clinical representation at case management and birth planning meetings for women in the care of substance misuse teams, and Prison, healthcare and nursery teams should hold regular management meetings to share information and ensure a joined-up, holistic approach • Breast feeding encouraged when safe • States that a substance misuse assessment must be completed, considering how to support both the family needs and substance misuse needs of the individual and how these can be managed on an MBU, prescribed medication for treatment of substance (includes prescribing guidance) use is permitted • Arrangements should be made for women to stay in hospital with their child or provide breastmilk if they are detoxing States that communication and management of women must be trauma-informed and responsive
Camden and Islington NHS Foundation Trust (2019)	Prescribing guidance for substance misuse services. [99]	• Philosophy of care / engagement • Practical clinical guidance / medical procedures • Prescribing protocols	• Although use of buprenorphine is not contraindicated, it is not recommended, as buprenorphine may induce a withdrawal state in the induction phase and put the pregnancy at risk (referenced); Does not recommend buprenorphine whilst breastfeeding, unless on specialist advice; pregnant women should not take dexamphetamine and naltrexone For benzodiazepine reduction maintain any existing methadone prescription—or buprenorphine—or gradual reduction advised on a long acting benzodiazepine • Discourage detoxification in the first trimester (risk of spontaneous abortion) and third trimester (risk of stillbirth); encourage breast-feeding unless using crack cocaine, cocaine or high dose benzodiazepines; Advise women test for HIV/Hepatitis • Multiagency working; liaise with other agencies; support women to attend antenatal appointments/social work case conferences • Balance the wish of the mother to be opiate free against the risk of withdrawal to the baby
Department of Health, Social Services, and Public Safety (2020)	Preventing Harm, Empowering Recovery: A strategic framework to tackle the harm from substance use (2021–31) [100]	• Overarching / organisational approach • Philosophy of care / engagement	• PWWUD/individuals in the pre and postnatal period may require additional support and alternative services • Need for bespoke services for women and girls; recognise that they experience increased rates of abuse and trauma, alongside concurrent stigma and barriers to accessing support. Recommends a trauma-informed approach and developing holistic and flexible joined-up services • Agencies should strengthen the links between maternity (inc. neonatal) and substance use services, and treatment services should work to make services more accessible for women and individuals with child-caring responsibilities • Recommends a values and evidence-based approach with a focus on shared responsibility, co-production of services and collaboration • Services should be universal, but with an increased focus on those most at risk • Recommends community-based treatment options with local flexibility to address needs and with a focus on long-term recovery and service delivery
Royal College of Obstetricians and Gynaecologists (2015)	Reducing the Risk of Venous Thromboembolism during Pregnancy and the Puerperium (2015) [101]	• Practical clinical guidance / medical procedures	• Risk assessment guidance categorises current intravenous drug users as 'intermediate risk' and the recommendation is to consider testing, supporting, and offering preventative treatment (prophylactic low-molecular-weight heparin (LMWH)) to women both antenatally and postnatally
Scottish Government (2018)	Rights, Respect and Recovery Scotland’s strategy to improve health by preventing and reducing alcohol and drug use, harm and related deaths. [102]	• Overarching / organisational approach • Philosophy of care / engagement	• Recommends generalised principles such as working in partnerships • Highlights the importance of challenging stigma; recognise that the whole family needs tailored, stigma-free support • Services should be informed by families lived experiences
Public Health England (2018)	Safeguarding and promoting the welfare of children affected by parental alcohol and drug use: a guide for local authorities [103]	• Overarching / organisational approach • Philosophy of care / engagement • Referral pathways • Child protection / safeguarding procedures	• Stresses organisations adopt a child-centred and whole family approach, joint working and potential for co-location of drug and alcohol and children services • Recommends joint working protocols, and information sharing agreements to be established between agencies • There should be clear referral pathways and procedures between drug / alcohol services and children's services, and other agencies • A trauma-informed approach should be developed and used in service delivery
Rotherham Safeguarding Children Partnership (2015)	Safeguarding Children of Drug Misusing Parents [104]	• Assessment • Child protection / safeguarding procedures • Overarching / organisational approach • Philosophy of care / engagement • Referral pathways	• Recognise that drug use is not always an indicator of significant harm; multidisciplinary assessment is necessary to ascertain need and level of risk/harm, including the impact on new-borns with NAS • Safeguarding guidance; any agency encountering a PWWUD, whose parenting capacity may be impaired must be referred to Children’s Social Care Services; If a newborn is withdrawing from substances at birth, a pre-discharge discussion, and potentially a Strategy discussion should occur before discharge • Hospital and community maternity services should be involved in all births in which a parent uses drugs • Information sharing should respect a pregnant woman’s confidentiality; agencies should share information sharing agreements • Women should be offered non-judgmental and supportive counselling and advice; services should be aware that PWWUD may present late to maternity/antenatal care, but that this could be an indicator of fear of stigma or service involvement, not necessarily of non-co-operation
Cumbria, Northumberland Tyne and Wear NHS Foundation Trust (2019)	Safeguarding Children Practice Guidance Note Addiction Services—Pregnancy Pathway and Guidance – V02. [105]	• Assessment • Child protection / safeguarding procedures • Philosophy of care / engagement • Practical clinical guidance / medical procedures • Prescribing protocols • Referral pathways	• Detailed clinical and procedural guidance on the roles, responsibilities and treatment / support to be offered by an allocated keyworker, pregnancy co-ordinator and medical staff, throughout the antenatal and postnatal period. It covers assessment, contact / appointments, information sharing, safeguarding, referral pathways and prescribing • It is based on ten principles: professionals delivering care will have appropriate skills and knowledge to deal with substance misuse in pregnancy; a Multi-Disciplinary Team (MDT) approach; PWWUD must receive the same quality of care, respect, and dignity as any other woman; a clear understanding of roles and responsibilities; a single plan of care agreed by the multidisciplinary team; addiction service will undertake an assessment and will become the main prescriber throughout, sharing details of the prescription with medical staff; the safety of the child will be paramount. effective communication and integrated working between agencies; a birth plan will ALWAYS be drawn up for child protection cases • Includes assessment and referral pathway flowcharts
South Gloucestershire Safeguarding Children Board (2015)	Safeguarding Guidance for Substance Misuse [106]	• Overarching / organisational approach • Philosophy of care / engagement • Assessment • Referral pathways • Practical clinical guidance / medical procedures • Child protection / safeguarding procedures	• Pre-birth assessments can provide opportunities for expectant parents to make positive changes; remind staff that most substance using parents want to be good parents, and that they are likely to be anxious about potentially losing their child; recommends equal treatment for all parents, and base judgments on evidence not optimism • Detoxification whilst pregnant requires specialist intervention; be aware of too-rapid detoxification or abstinence carry risk of relapse; do not assume that abstinence will improve parenting; encourage antenatal attendance where possible • Children born to PWWUD will require ongoing follow-up and monitoring for special health needs; recommends continuous assessment every 6 months post-birth • If a PWWUD is under 18 years old, services must engage with young people’s drug/alcohol agencies • Includes safeguarding and referrals flowchart
Rotherham Safeguarding Children Partnership (2016)	Safeguarding Unborn and Newborn Babies [107]	• Overarching / organisational approach • Assessment • Referral pathways • Child protection / safeguarding procedures	• Referral to children's services to be made ASAP • Detailed guidance around a pre-birth strategy discussion, assessment, and conference. The Pre-birth Triangle is presented as a model for assessing risk to unborn baby and parenting capacity. All agencies to be involved in assessment / strategy discussion • Following pre-birth Assessment, an Initial Child Protection Conference should be held as early as possible with a Child Protection Review Conference held at least 6 weeks before the expected birth • A clear action plan to be created to include actions for all agencies involved—and this to be communicated with parents. If significant risk identified a pre-birth child protection case conference to be held, and decision made about care of baby after birth. If baby to be removed from mother’s care—protocol on legal requirements, and communications with parents. This includes arranging support for parents • Sets out what must be considered at a prebirth and post birth discharge planning meeting; this includes planning contact arrangements if separation planned • Flow charts provided to cover the full safeguarding process and responsibilities / tasks at each point
NHS England (2019)	Saving Babies Lives Care Bundle v.2 [108]	• Assessment • Referral pathways • Practical clinical guidance / medical procedures	• Offers guidance related to drug misuse as a moderate risk factor in Foetal Growth Restriction (FGR): • Prevention = Assess for history of placental dysfunction and consider aspirin 150mg at night < 16 weeks as appropriate • Risk Assessment/Triage Pathway = Anomaly scan and Estimated Foetal Weight ≥ 10th percentile • Surveillance Pathway for FGR = Serial ultrasound scans from 32 weeks every 4 weeks until delivery • Ongoing assessment for complications developing in pregnancy e.g. hypertensive disorders or significant bleeding • Advise women to tell their midwife about any illegal drug use; encourage/reassure them that any disclosure will be treated with confidence, and information only shared with relevant healthcare professionals • Advise women to contact FRANK (drugs advice service, via phone)
Shetland Child Protection Committee (2017)	Shetland Integrated Children's Services Plan [109]	• Overarching / organisational approach • Philosophy of care / engagement	• Promotes a child centred, family-based approach; focus on early intervention and prevention • Recommends a lead professional identifiable to family • Non-judgemental approach, and that professionals use a holistic multi-agency approach to assess and respond to need within each individual situation • Information sharing and joint working between agencies
Southwark Safeguarding Children Partnership (2020)	Southwark Joint Service Protocol to meet the needs of children and unborn children whose parents or carers have substance misuse problems [110]	• Overarching organisational /approach • Philosophy of care / engagement • Assessment • Referral pathways • Practical clinical guidance / medical procedures • Child protection / safeguarding procedures	• Agencies are responsible for identifying PWWUD who may be in need of additional support services; PWWUD are more likely to experience difficulties during pregnancy and following the birth of their baby • Assessments should be conducted when substance use is identified, to determine other service requirements; including information from their GP, substance misuse services, social services, other agencies etc. and details of previous diagnoses, treatment history and co-existing social problems; liaison with social care regarding previous births is essential; refer PWWUD who have uncontrolled use to specialist services • Substance use services should provide multi-agency support for PWWUD • A pre-birth assessment should be undertaken once drug use in pregnancy is identified • Cessation/reduction in substance use should only be undertaken following consultation with midwifery service/key worker in substance misuse services • New-born's clinical presentation may require a referral to specialist substance misuse antenatal services, and dispute/lack of referral clearly documented • Antenatal services should be accessible, and provide honest, sympathetic, consistent and non-judgemental care to help alleviate feelings of guilt/anxiety/being stigmatised • Advises that women informed of the risks can remain on methadone/buprenorphine during pregnancy; “There is no good evidence of benefit derived from substitution therapy with benzodiazepines during pregnancy”, but notes this could be considered in exceptional circumstances; majority of cases, PWWUD should attempt reduction in use under specialist advice • Encourage breastfeeding in cases where drug use is stabilised • Includes detailed referral pathway and decision-making flowchart
NHS Greater Glasgow & Clyde Alcohol and Drug Recovery Services Pharmacy Team (2019)	Standards for Drug & Alcohol Services in Community Pharmacies [111]	• Prescribing protocols	• Promotes harm minimisation • Objective is to stabilise the woman. Detox should only be conducted if it is considered to be appropriate. If requested, not to be considered until 2nd trimester. Pregnant women to remain on current treatment, methadone, or buprenorphine • Transfer to buprenorphine during pregnancy is not usually advised because of the risk of triggering withdrawal and the risk of inducing withdrawal in the foetus
North Yorkshire Safeguarding Children Partnership (2019)	Substance Misuse in Parents [112]	• Overarching / organisational approach • Philosophy of care / engagement • Assessment • Referral pathways • Child protection / safeguarding procedures	• Recommends multi-agency working and collaboration between children/family services and substance misuse services to identify, assess, support and treat adults whilst protecting children • Joint working and sharing information is recommended—drug testing as part of substitution therapy, with results shared if necessary in child protection contexts • The needs of children must always come first; Notes that not all parents or carers with drug or alcohol problems cause harm to children in their care, but substance use can reduce capacity for effective parenting • Midwife should be the main point of contact for all agencies, taking the lead on information sharing and co-ordinating service provision for PWWUD whilst ensuring the woman is fully informed • Substance misuse and Children and Families service support plans should reflect a holistic approach to assisting families
NHS Lothian—Anne Whittaker (2003)	Substance Use in Pregnancy [113]	• Philosophy of care / engagement • Assessment • Referral pathways • Practical clinical guidance / medical procedures • Child protection / safeguarding procedures	• Stresses that many factors affect pregnancy and substance misuse is just one • Recommends a holistic assessment, and that workers have a non-judgemental, pragmatic approach, emphasising harm reduction. Focus on women's needs and choices • A co-ordinated multi-disciplinary approach, with clear roles and responsibilities of each agency is recommended • Drug treatment approach to be realistic and tailored to individual • It is important that PWWUD follow clear care pathway: includes detailed timeline of information to be provided, assessments, risk assessments, birth and post-partum care planning etc • Women are advised to remain in hospital for 72 h post-birth for monitoring of NAS • Child protection assessment and referral procedures includes clear criteria for concerns / risk as well as strengths and protective factors, and early intervention strategies that can support the woman in her parenting • Child protection case conferences should be held 6–8 weeks before the birth date. (32 weeks) • Includes detailed week-by-week schedule of care
Hull Safeguarding Children's Partnership (2022)	Substance Misuse in Pregnancy [114]	• Overarching / organisational approach • Philosophy of care / engagement • Assessment • Child protection / safeguarding procedures	• PWWUD should be encouraged to access antenatal care and treatment early; primary focus is on supporting women, but partners and wider family need to be accounted for • Respect confidentiality and explain the importance of information sharing; care should be non-judgmental, non-stigmatising and receptive • At least one (ideally the first) midwifery appointment should be one-on-one, and early identification of vulnerabilities is essential, including risk factors that contribute to poorer outcomes in children; consider and promote protective factors
University of Leicester Hospital Trust (2019)	Substance Misuse in Pregnancy – Guidance for the care of pregnant drug /alcohol users and their babies. [115]	• Philosophy of care / engagement • Assessment • Referral pathways • Practical clinical guidance / medical procedures • Child protection / safeguarding procedures	• Comprehensive guidance on the practical steps for assessing and supporting PWWUD at each stage of pregnancy, during and after birth • Includes referral and care pathway flowchart and detailed schedule of care • PWWUD to be referred to specialist multi-disciplinary substance misuse team, substance misuse midwives to complete assessment for which there a range of potential outcomes described including a brief intervention / continuation of care by community midwives / specialist midwives to address risks posed by substance misuse etc • Labour and birth recommendations include continuation of methadone / substitution therapy; all staff to be aware of women's drug use; women to be on open ward; pain relief to be administered PRN; infant to be observed for NAS for 72 h • Clear guidelines on procedure if baby develops NAS; recommends breast feeding, and management to be conducted in a holistic, family-centred way • Discharge only to be planned once any case conference etc. held, and social situation clarified • Threshold for referral to children's services recommended to be low, both antenatally and as inpatient
Heart Of England NHS Trust (2016)	Substance Misuse in Pregnancy (V.4) [116]	• Overarching / organisational approach • Philosophy of care / engagement • Assessment • Referral pathways • Practical clinical guidance / medical procedures • Child protection / safeguarding procedures	• Includes flowcharts and schedules of care which outline steps to be taken at each stage which outline instructions for staff liaisons, scans and ultrasounds as necessary; screening in pregnancy and labour begins with testing of urine, and referral to specialist substance use midwife • Antenatal care; ask women sensitively about substance use; non-judgemental care encourages antenatal attendance, improves monitoring capabilities, and contributes to better outcomes for the baby; Domestic Abuse screening—women to be asked directly/sensitively about injuries (with a non-family interpreter if necessary); • Women should be referred if not in treatment to specialist services for in-depth assessment including ongoing counselling and stabilising use through substitute prescribing; antenatal care should be considered a key time to address substance misuse; assessments of women’s social circumstances should also be made; if safeguarding concerns arise, refer onto safeguarding midwife • Mothers to be screened for blood borne viruses, and tests to be offered; mothers that are HIV positive to be referred to specialist care • Recommendations for NAS—women to be given written/oral information and made aware of care pathways; babies at risk of NAS are not to be delivered at Solihull; keep mothers and babies together where possible; only separate as a last resort; if a mother is only allowed supervised access, the baby must be accommodated on the Neonatal Unit; hospital staff cannot continuously monitor mothers with their babies on postnatal wards; women should be encouraged to remain in hospital for a minimum of 72 h to be monitored for NAS • Recommendations for inpatient care include ensuring adherence to methadone prescription and thoroughly investigating claims dose has been missed • Recommends breastfeeding unless woman is HIV positive • Post birth—Child Protected Case conference should be organised if there is a history of previous children removed into care; facilitate mother: baby bonding; assess maternal wellbeing and parenting skills; report child protection concerns; discharge planning meeting with multi-disciplinary team • Discharge planning involves; notifying a woman’s specialist midwife/drugs worker; recording urine for medical records; ensuring OST prescriptions are in place and accessible
Walsall Healthcare NHS (2017)	Substance misuse in pregnancy and subsequent care of the newborn [117]	• Referral pathways • Practical clinical guidance / medical procedures • Prescribing protocols	• Women who are identified as using drugs during pregnancy to be referred to drug treatment services, if not already involved, children's services, and consultant care for maternity to support multi-disciplinary working. To be encouraged to engage with antenatal care • Detox from drugs not encouraged—try to stabilise the women on methadone / buprenorphine. This to be continued to be given throughout pregnancy and labour, and additional pain relief prescribed if required • Women to be made aware of risk of NAS, stay on ward for 72 h after birth to observe baby for NAS • Breast feeding to be encouraged except known cases of HIV • Upon discharge, care planning meeting with social care to be held, and community drugs team informed • Includes Antenatal care flowchart
Royal Cornwall Hospitals NHS Trust (2020)	Substance Misuse in Pregnancy, Labour and Post Delivery Clinical Guideline [118]	• Overarching/ organisational approach • Philosophy of care / engagement • Referral pathways • Practical clinical guidance / medical procedures • Prescribing protocols • Child protection / safeguarding procedures	• All women to be asked at booking about substance use and advised it is safest not to; if drug use is identified, referred to addiction services, screened for Hepatitis, initial safeguarding paperwork completed. Information will be shared between safeguarding midwife/addictions worker/social worker; if partner is using, encourage them to seek support from addiction services Methadone / buprenorphine to be prescribed as required from admission throughout pregnancy • Refer to Multi Agency Referral Unit if regular antenatal non-attendance occurs, involving other professionals care for the woman • If safeguarding paperwork has commenced, these women are to be discussed at monthly vulnerable women’s meetings • Safeguarding midwives must be informed upon antenatal admissions and intrapartum care • Baby to be monitored for 72 h for NAS; breastfeeding is encouraged • Upon discharge, prescriptions of methadone/Subutex must be provided; where there are safeguarding issues on discharge, discharging midwife should contact safeguarding midwife, health visitor and social worker; safeguarding midwife is the designated lead for monitoring and compliance • Includes substance misuse assessment and referral pathway flowchart
Frimley Health NHS Foundation Trust (2021)	Substance Misuse in Pregnancy: multidisciplinary guidelines for Frimley Health NHS Foundation Trust [119]	• Philosophy of care / engagement • Assessment • Referral pathways • Practical clinical guidance / medical procedures	• Key messages are to work in partnership with parents and adopt a non-discriminatory / non-judgemental approach • Multidisciplinary working in both the antenatal and postnatal period is imperative • Detailed outline of the roles and responsibilities of key healthcare staff: midwives, crystal / orcid team, and obstetricians • Recommends mother remains on the ward for a minimum of 72 h, as withdrawal symptoms may present up to four days post-birth
Welsh Government (2011)	Substance Misuse Treatment Framework (SMTF) Guidance for Evidence Based Community Prescribing in the Treatment of Substance Misuse [120]	• Overarching / organisational approach • Assessment • Referral pathways	• Recommends ongoing communication and information sharing between specialist substance misuse and midwifery/obstetric team • Assessment of risk should occur as soon as possible in the pregnancy, to develop integrated support networks and care plan • Communicate necessary information for women to support informed decision making • Monitor dosages closely to ensure adherence to prescribing compliance; perform routine toxicology testing • Ideally, specialist midwife should coordinate care and signpost to other services • Outlines comprehensive assessment guidance; identify the nature and severity of the problem and issues around substance misuse; explore the reason(s) for misuse; assess the impact of substance misuse on an individual’s physical, psychological and social functioning; ascertain the client’s cognitive ability; establish the personal resources individuals have to deal with treatment, including support from their family and friends
Birmingham Women and Children's NHS Foundation Trust (2021)	Substance Misuse: Management of Pregnant Women [121]	• Practical clinical guidance / medical procedures • Referral pathways • Prescribing protocols • Child protection / safeguarding procedures	• PWWUD to be referred to specialist midwives who will provide assessment. Outcome could be referred to community midwives, or care to be provided by specialist midwives. They provide specialist care / advice, perform random drug screening to ensure compliance with drug treatment programme, share information with other professionals including community midwife, and drug treatment services • Recommends referral to the Birmingham Safeguarding protocols, and if significant concern for safety of unborn baby / infant then a Request for Support Referral to Children’s Social Care should be made, preferably with consent • Labour—should be no different than any other women and follow the women's preferences—any substitute treatment they take to be continued to be given • Postnatal care—To be transferred together to ward—for women using opiates—observation for 72 h for NAS—which should have been discussed with women in antenatal period. Breast feeding to be encouraged—unless chaotic drug use / HIV positive • Detailed prescribing advice / recommendations provided throughout in relation to OST, women continuing to use illicit substances etc. Recommends women are stabilised on methadone, and that a flexible approach to OST prescribing is adopted. Advises against prescribing Subutex stating that it is widely known to cause NAS • Full timeline of checks / assessments to be done, referrals to be made, information to be provided, and information to be shared • Includes referral pathway/assessment flowcharts
Public Health England (2013)	Supporting information for developing local joint protocols between drug and alcohol partnerships and children and family services [122]	• Overarching / organisation approach • Philosophy of care / engagement • Assessment • Referral pathways • Child protection / safeguarding procedures	• Service users should be asked about pregnancy; the risks associated with drinking during pregnancy should be discussed • Local protocol should outline arrangements for working with PWWUD (inc. partners and family); wider alcohol screening in antenatal services could be considered; explore potential for specialist interventions (e.g. Family Nurse Partnerships); early access to antenatal care/joint care planning should be promoted through local arrangements • Treatment providers should maximise the potential for pregnancy to facilitate motivation to change behaviour • Refer PWWUD to specialist midwives where possible; referral pathways/criteria for referral should be specified; where no specialist midwife is available, arrangements for management of OST should be set out • Flowchart of action for referrals to children/family services; pregnant service users to be referred to antenatal care for assessment of treatment/support needs, and detailed week-by-week schedule of care
Tayside Multi-agency Partnership (2021)	Tayside Multi-Agency Practitioner’s Guidance: Concern for Unborn Babies [123]	• Child protection / safeguarding procedures	• Overall encourages information sharing between agencies in the best interest of unborn baby / infant / child and explaining to the families the reasoning behind sharing. Recommends all staff read, are informed of and follow Scottish Government child protection policy documents, and reports relating to parental drug / alcohol use • Outlines risk of harms to unborn baby / infants. Recommends practitioners understand when to share information; what to share; how much to share; who to share information with and how to share. They must also understand the potential risk of harm if they do not share information • Desired outcomes include effective inter/intra agency working, holistic assessment and co-ordinated joint working throughout pregnancy and immediately following birth
NHS Ayrshire & Arran (2019)	The Management of High-Risk Pregnancies [124]	• Assessment • Referral pathways • Practical clinical guidance / medical procedures • Child protection / safeguarding procedures	• Outlines the referral/assessment process; identified substance use (high risk criteria) necessitates referral to specialist midwife within 2 working days; specialist midwife to conduct pre-birth assessment; results shared with child health protection teams/other necessary agencies, including police to conduct background checks • Consent is not required to share information in the pre-birth assessment, but is good practice • The child protection health team is responsible for compiling relevant agency assessments into a master assessment form • States that pre-birth Child Protection Case Conference (CPCC) should take place no later than at 28 weeks pregnancy • Includes flowcharts related to referrals and care pathways
National Collaborating Centre for Mental Health (2018)	The perinatal mental health care pathway; Full implementation guidance [125]	• Overarching / organisational approach • Philosophy of care / engagement	• Whole systems approach to the provision of care; multi-disciplinary • Emphasises a recovery-based approach to be taken • Co-ordinated care plan to be created together with the woman, and her needs to be at the centre • Includes detailed schedules of care, and recommends a biopsychosocial/holistic assessment, considering multiple needs
Norfolk & Waveney NHS Trust (2021)	Trust Guideline for the Care of Vulnerable Women in Pregnancy [126]	• Overarching / organisational approach • Philosophy of care / engagement • Assessment • Referral pathways • Child protection / safeguarding procedures	• Identification of risks, assessments and care planning should follow the general guidance regardless of vulnerability; women who disclose substance misuse at booking should be referred to appropriate specialist services and consultant led antenatal care • If safeguarding concerns arise, the pre-birth protocol is to be initiated; all women with safeguarding concerns to receive joint visits with a name health visitor, • Good communication is essential in multiagency working, between agencies, specialist services, maternity services and the women; communication should always been sensitive and confidential • All agencies and specialist services engaged with a PWWUD should be informed of a delivery; continued assessment to be provided by the community midwife up to 28 days postnatally, where appropriate • Notification of discharge from maternity services should be made to relevant services and agencies, including the named health visitor; women and their babies should receive ongoing support and assessment • Women should be aware of all relevant contacts (Medicom/GP/Health Visitor/out of hours services); change of addresses/contact details is imperative • Women who do not want to continue a pregnancy are to be referred to GP/local termination services; if a woman is undecided, referral to BPAS for advice and counselling if appropriate
Hull Safeguarding Children's Partnership (2022)	Unborn Procedures and Guidance (Pre-Birth Pathway) [127]	• Overarching / organisational approach • Referral pathways • Child protection / safeguarding procedures	• Midwives complete a Pre-Birth Vulnerability Screening Tool—drug / alcohol use is a recognised category of vulnerability and triggers early referrals to specialist agencies • All agencies to contribute to pre-birth assessment if one is needed. Information sharing is crucial • Step—Up / Step down approach to safeguarding interventions—If no significant risk identified but need for support is then refer to Targeted Early support team or Significant harm pathway into Children's Social Care—where risks are identified. If risks relate to substance misuse it must be clearly specified what these are • Multi-disciplinary planning meeting—to be held no later than 20wks, all agencies who will contribute to pre-birth assessment or care plan to contribute. Any assessment conducted to be shared with family by 36 weeks • Birth plan to be shared with safeguarding midwife. If Child protection Case Conference needed this should be held before 32 weeks • Staff doing home visits are urged to be aware of need to assess parenting capacity and household environment etc
Scottish Government (2015)	Universal Health Visiting Pathway in Scotland: Pre-birth to Pre-school [128]	• Assessment • Referral pathways	• Overall, health visitors should work to support a reduction of parental substance misuse where identified; discussing the risks involved with substances on individuals and babies health; make relevant referrals to cessation services • Provide parenting advice with a focus on attachment—reducing substance misuse is a key parenting issue; substance misuse should be discussed and assessed continuously • Includes a detailed schedule of a pregnancy and the relevant care delivered during each time frame • Notes that Health Visitors work holistically with families
East Ayrshire Child Protection Committee (2017)	Vulnerable Pregnancy Procedure [129]	• Assessment • Referral pathways • Child protection / safeguarding procedures	• Multi-agency working; person centred, and needs led assessment and care-planning • If concerns for vulnerable pregnancy (substance misuse is recognised category) referral should be made to initial response team. Community midwife should make referral to safeguarding midwife (SGM). SGM notifies Child protection Health Team • Multi-agency discussion to be held if vulnerable pregnancy and no SW involvement; decision to be made about lead agency; case to be referred to MARG • Pre-birth assessment to be completed by SGM, and shared as appropriate / or community midwife if this was agreed • A child protection pre-birth conference should take place on or before the 28th week pregnancy, or within 21 days for a late presenting pregnancy • Includes referral flowcharts • Child Protection concerns identified at any stage a CP alert raised. If concern established SW to lead. If CP case conference held—should decide on what must happen re: care of infant following birth. If no concerns case reverts to community midwives
West Yorkshire Consortium Inter Agency Safeguarding and Child Protection Procedures (2022)	West Yorkshire Consortium Inter Agency Safeguarding and Child Protection Procedure 1.4.15 Children of Drug Misusing Parents [130]	• Overarching / organisational approach • Assessment • Referral pathways	• If a PWWUD is identified, referrals must be made to Children’s Social Care Services, and a Pre-Birth Assessment initiated • Recommends following the Care Planning Approach / Care Co-ordination procedures, which includes input from the link midwife and social worker from Children’s Social Care Services • Notes that all maternity services should have procedures for pregnant women who use drugs that encourage them to access antenatal services which will help them stabilise, reduce or stop their drug use; PWWUD should be encouraged to contact the Substance Misuse Team for assessment and treatment options • If a newborn is found to be needing treatment for NAS, a pre-discharge should be scheduled and potentially a Strategy Discussion pre-discharge
Highland Council & NHS Highland (2019)	Women, Pregnancy and Substance Use: Good Practice Guidelines [131]	• Philosophy of care / engagement • Referral pathways • Practical guidance / medical procedures • Prescribing protocols • Child protection / safeguarding procedures • Specific recommended interventions	• Emphasis on holistic assessment of need, multi-agency working, engaging women in specialist services early in pregnancy using sensitivity and promoting the women's autonomy. All staff to adopt a trauma informed, non-judgemental and empathic approach. Staff to be open and honest about their role and responsibilities • Full schedule of maternity care, and support to be provided at each stage. All staff to be trained in supporting PWWUD. Antenatal Plan: additional support for mother and unborn baby’ should be completed by the named midwife—detailing any identified harms to the unborn baby or mother. Early referrals and interventions recommended • PWWUD to follow RED pathway of care—to be reviewed by obstetrician and individual care plan by maternity care team • Includes referral flowcharts, recommends women to be referred to specialist drug treatment and support service • Information to be shared between agencies—clear lines of communication • OST to be prescribed throughout. Recommends prescribing Buprenorphine stating that there is evidence of better neonatal outcomes. States that methadone has been linked to visual disorders in infants. If a woman is already being prescribed methadone, she can choose to continue with this prescription following discussion around potential risks • Women who have been using drugs, or on a substitute prescription are expected to remain in hospital for 5 days, as NAS may occur later • A pre-birth planning meeting must take place no later than 28 weeks gestation following any concerns of substance use • Motivational interviewing identified as positive tool to engage women
UK Government (2018)	Working Together to Safeguard Children A guide to inter-agency working to safeguard and promote the welfare of children [132]	• Assessment • Referral pathways • Practical clinical guidance / medical procedures	• Practitioners should be alert to the potential risks of drug use in parents, other guidance regarding multiagency assessment, early help, referral and information sharing are generic to all children and safeguarding •Includes flowchart for multiagency referrals/assessment/procedures • Notes that a high-quality assessment for a child will be holistic
Derbyshire Safeguarding Children Board (2022)	Working with parents who are misusing substances [133]	• Assessment • Referral pathways • Child protection / safeguarding procedures	• Any concerns re: substance misuse should be shared with midwife. Promotes information sharing, joint assessments and care planning between agencies Early Help or pre-birth assessments to be conducted where there is concern about parenting capacity • Advocates for testing for illicit drugs even if women taking OST • If NAS occurs unexpectedly should talk to women about what substances illicit or otherwise, she took during her pregnancy. If illicit, referral to children’s services • A multi-agency Pre-Discharge meeting should be held—clear multi-agency care plan for ongoing assessment, monitoring and support
Aberdeen City Child Protection Committee (2017)	Working with vulnerable unborn babies and their families multi-agency practice guidance [134]	• Overarching / organisation approach • Philosophy of care / engagement • Assessment • Referral pathways • Practical clinical guidance / medical procedure • Child protection / safeguarding procedures	• Overarching principles; Rights of the child to be safe are paramount • Working alongside parents in partnership is vital; clear criteria around information sharing and what can remain confidential when considering a child’s safety; assess parenting/caring skills of fathers/significant male figures rather than solely on mothers; Assess the risks of significant parental drug use may have on children/young babies • Be aware of complex mental health difficulties in children, which may require comprehensive multi-agency assessment; be aware that learning difficulties/complex health challenges may impact on parental caring capacity; building trusting relationships between professionals/parents is imperative • Be aware of the risks of domestic abuse; be alert to potential harm violent men can inflict on women and children; take account of who is living in households with children, and who might be in contact • Multi-agency collaboration; police records show past criminal convictions/activity to be used in risk assessment; obtain as much information about parent’s childhoods to identify sources of resilience/parenting styles; Inter-agency collaboration between adult & child services is recommended; • Advocates for an ecological approach to be taken (child development is seen ‘in context’); assessment should be holistic and consider the whole picture, inc. other services involved, and who should the family be referred on to? • Agencies should not advise abstinence or cessation from drugs without GP/midwifery/substance use service advice; similarly, should not advise stopping psychiatric medications without appropriate medical consultation • Includes Referral pathways flowchart
World Health Organization (2014)	Guidelines for the identification and management of substance use and substance use disorders in pregnancy [135]	• Philosophy of care / engagement • Assessment • Referral pathways • Practical clinical guidance / medical procedure • Specific recommended interventions	Overarching principles 1. Prioritizing prevention 2. Ensuring access to prevention and treatment services 3. Respecting patient autonomy 4. Providing comprehensive care (matching complexity of substance use disorder) 5. Safeguarding against discrimination and stigmatisation • Recommends screening for substance use, and a brief intervention be offered to all women using drugs/alcohol • Healthcare providers should offer comprehensive assessment and individualised care, which includes care that is responsive to multiple needs including family/relationships, other medical needs, housing and poverty and violence (holistic) • At the earliest opportunity, recommend cessation of drug use (with appropriate detoxifications services/referrals), with exceptions made for opioid/benzodiazepine use, in which case opioid management treatment, or gradual dose reduction of benzodiazepines using long-acting benzodiazepines is recommended • In cases of stimulant dependence, psychopharmacological medications are not routinely required, but may assist with symptoms of psychiatric disorders • Opioid maintenance therapy is recommended (either methadone or buprenorphine) in combination with psychosocial intervention • Breastfeeding is encouraged, unless specific risks are present (HIV status), and skin-to-skin contact actively encouraged regardless of feeding choice • Healthcare facilities should have scope/facilities for assessing and treating babies exposed to opioids. If necessary, opioid treatment should be used for exposed infants, and if withdrawal occurs from sedatives or an unknown substance, phenobarbital may be the best initial treatment • Infants exposed to opioids should remain in hospital for 4–7 days and be monitored for withdrawal symptoms with a validated assessment instrument • Notes that woman-centred, trauma informed care which includes pharmacotherapy is best-practice but is also the costliest approach

### Characteristics

#### Geographical coverage and intended users

The four UK nations have their own health care systems, with independent policies and guidelines in addition to taking lead from NICE (a UK wide executive public body funded by UK Government, which provides guidance, advice, quality standards and recommendations relating to health and social care, including clinical practice). The documents included in this review covered a range of geographical areas and were written for a variety of intended users. One international document applicable within the UK was identified [135], 26 documents were UK-wide, 20 were specific to Scotland, or Northern Ireland or England and Wales, and one document was specific to Wales (Table 3).

Documents were designed to be implemented by a wide range of users (maternity staff, healthcare professionals, social workers, substance use service staff and pharmacists etc.), with some relevant to more than one user group. Notably, 14 documents stated they could be used by patients or service users (Supplementary Table 4).

#### Setting

Approximately half the documents were applicable in any setting where a professional may be supporting a pregnant woman who is using, or in treatment for drug use (*n* = 60). Some were specific to hospital care (*n* = 21), or for use within a community setting (*n* = 13) (including community midwifery services, community pharmacies and substance treatment services) (Table 3).

#### Relevance

Most included documents were relevant across the whole perinatal period (*n* = 85), with 17 also applicable to families throughout childhood, for example, child protection and safeguarding guidance. Twenty-three were relevant to women during pregnancy, and only three were specific to the postnatal period (Table 3).

The relevance of documents to pregnant women who use or are in treatment for drug use, and their babies varied; some were entirely specific to drug use during the perinatal period, whilst others included specific sections or brief mentions for the population. Universally relevant documents were also identified, which provided overarching guidance and directives for groups and individuals on a broader topic (such as vulnerable pregnancies) but included pregnant women who use or are in treatment for drugs within its scope (Supplementary Table 5). Forty-four of the documents specifically referred to this population as vulnerable (Table 3).

#### Evidence base

Documents varied in their development methods and extent to which they were evidence-based. Just over half (*n* = 61) were unclear about development methods, with the remainder outlining approaches including conducting a review of evidence, expert opinion or consultations with service users or with the public. Ten documents used all three methods, with the remainder employing one or two of the approaches (Supplementary Table 6).

While twelve documents did not report drawing on any form of evidence, most cited between one and three different types of evidence, and twenty-six referenced between four and six different types of evidence (Supplementary Table 6). The most common form of evidence cited was other guidance documents (including NICE and SIGN guidance, reports, policies, and strategies).

### Recommendations

#### Overarching/organisational approach

Guidance relating to an overarching organisational approach (Table 5) focused on multi-agency working and systems to support this, including information sharing, collaboration, and shared care-plans. Recommendations for organisations included developing and delivering training [43, 90, 91], asking women about the acceptability of services and co-producing local services with women [57], increasing maternity staffing levels including specialist midwives [91], creating a working group [91], and improving recording and monitoring systems in maternity care [57] (Table 4 and Supplementary Table 6).

**Table 5:** Categories of best practice recommendations

Category of best practice recommendations	Description
Overarching / organisational approach	Related to ways that services are delivered, organised and / or commissioned. Statements aimed at service commissioners and providers
Philosophy of care / engagement approach	How practitioners should approach / work / engage with the women
Assessment	Relates to all forms of assessment in all contexts
Referral Pathways	Refers to guidance around who women / babies should be referred onto, and circumstances around this
Practical clinical guidance / medical procedures	Instructions relating to the treatment of patients and medical procedures to be carried out
Prescribing protocols	Relates to guidance around prescribing opioid substitution therapy (OST), withdrawal, and detox
Child Protection / safeguarding procedures	Covers risk assessment, care-planning, interventions, and any other recommendations related to child protection or safeguarding
Specific recommended interventions	Specific intervention models or tools

#### Philosophy of care and engagement

Guidance relating to how practitioners’ approach and care for pregnant women who use or are in treatment for drug use (categorised as philosophy of care / engagement approach; Table 5, Table 4 and Supplementary Table 6) was identified in over half of included documents (*n* = 64).

Overall, a non-judgemental, sensitive, and respectful attitude to women who use or are in treatment for drug during pregnancy was recommended. There was also advice around understanding and considering the woman’s wider circumstances, and family situation. Adopting a trauma-informed approach was specifically mentioned in more recent included documents (*n* = 21) and was first identified in the WHO (2014) guidance. Some of the child protection and safeguarding procedures suggested staff should be mindful that drug use is only one factor affecting pregnancy and does not necessarily mean parents are unable to provide ‘good enough’ parenting or a child is at serious risk of harm (Table 4 and Supplementary Table 6).

The NICE guideline, ‘Pregnancy, and complex social factors’ [95] recommended consistency and continuity of care, together with offering a flexible approach to support the engagement of pregnant women who use or are in treatment for drug use, as they may find engaging with services difficult. This way of working was echoed within many other included guideline documents, with references to the NICE guideline [95] (Table 4 and Supplementary Table 6).

Additionally, documents included discrete recommendations for care approaches such as using a care planning / care-coordination approach [46, 48, 130], utilising a recovery-based approach [125], and advocating for prevention and early intervention [49, 109, 113]. Training to ensure practitioners understand the complex needs of pregnant women who use or are in treatment for drug use was suggested in five documents [43, 75, 90, 91, 95].

### Assessment

Guidance for the assessment of pregnant women who use or in treatment for drugs addressed the period from the maternity service antenatal booking appointments, pre-birth child protection and safeguarding assessments through to labour pain management and hospital discharge arrangements. Across all types of documents, some consistent practice recommendations were identified, including the importance of assessing and asking questions about substance use and not assuming other professionals will. There were also specific tasks to be completed such as screening for blood borne viruses.

A multi-agency approach to assessment, with an identified lead agency was preferred. It was commonly suggested that assessment should consider the wider social, emotional, and practical needs of the individual (for example access to housing etc.). Many documents also stated fathers or partners should be included in assessment, with some suggesting a family-based approach be taken.

Risk assessment was described as an on-going process that should be continuous throughout pregnancy. Most documents focused on the risk to the unborn or new-born baby, with the safety of the child as the central concern (See Child Protection / Safeguarding Procedures below).

A range of assessment tools were identified including: SHANARI Wellbeing Assessment tool [83]; Pre-birth triangle [107]; Pre-birth Vulnerability Screening Tool [127]. Many documents also included specific assessment flow charts within their appendices (Table 4 and Supplementary Table 6).

### Referral pathways

Clear referral pathways for pregnant women who use or are in treatment for drug use were recommended in many documents (Table 4 and Supplementary Table 6). Flow charts outlining referral pathways were often included within appendices. Most documents recommended a referral to specialist midwife services (substance use; additional needs and vulnerabilities; safeguarding) and drug services both for women and their partners if not already engaged in drug treatment. Referrals to mental health and counselling services for women were also recommended. Most documents that covered assessment and care planning, outlined when referrals should be made to children’s social care / child protection services (see Child Protection / Safeguarding Procedures below).

### Prescribing guidance

Of the included documents, eight were specifically focused on prescribing guidance for women who use opioids, whilst others included sections or reference to this. Overall, suggested practice was to refer women who use opioids to drug treatment services for assessment and opioid substitution treatment (OST), which recommendations stated should be prescribed throughout pregnancy, including during labour.

Although one recently published guideline [131] stated buprenorphine has been shown to have better neonatal outcomes than methadone, citing evidence of visual impairment in infants exposed to methadone prenatally, most documents suggested methadone is preferred over buprenorphine during pregnancy. Most documents advocated for OST as part of a harm reduction approach although one document suggested “*Abstinence can be helpfully thought of as the ‘final goal’ of harm reduction*” [42] appearing to challenge the idea of harm reduction as a legitimate alternative to abstinence from OST.

Where detox was deemed medically necessary, most documents advised not detoxing women in their third trimester (due to associated risks) and suggested if detoxification from opioids was conducted, it should be during the second trimester in small, frequent reductions. There were some recommendations for in-patient opioid detoxification with women’s informed choice and managed detoxification from benzodiazepines and cocaine (Table 4 and Supplementary Table 6).

### Clinical and practice guidance

Many documents were clinical guidelines or contained practice guidance specific to pregnant women who used or were receiving treatment for drug use, and several contained a step-by-step guide outlining the treatment and care to be provided at each point throughout pregnancy (Table 4 and Supplementary Table 6).

Practice guidance included encouraging women to access antenatal care, the provision of information on the effects of drugs and alcohol on the fetus and the risks of neonatal abstinence syndrome. There were also specific suggestions to measure the abdominal circumference of women using benzodiazepines at 28–30 weeks, and 32–34 weeks [27] in order to monitor for intrauterine growth restriction (IUGR) [136]. There were some recommendations to conduct drug testing at booking, throughout pregnancy, and in labour, with different documents recommending differing time scales for this (Table 4 and Supplementary Table 6), and one suggestion that urine testing should be supervised [26].

In general, the documents suggested standard intrapartum care should apply, with guidelines concerning pain relief during labour recommending women should be prescribed pain relief as needed, regardless of whether they were receiving OST. Most recommended women should continue to have access to OST while in hospital, and there were detailed protocols for sharing prescribing information between drug services, maternity staff, anaesthetists, and hospital pharmacists, including arranging for provision if, for example, women were admitted to the labour ward at the weekend (Table 4 and Supplementary Table 6).

Recommendations for a woman and her baby to be observed for Neo-natal Abstinence Syndrome (NAS) in hospital were identified in 14 documents, with lack of consensus regarding the length of post-birth monitoring varying from 72 h (*n* = 9), four days (*n* = 1) [32] and 5–7 days (*n* = 4) [45, 67, 77, 131]. Overall, documents recommended breastfeeding was to be encouraged unless the mother was using cocaine, was HIV positive, or on high doses of benzodiazepines (Table 4 and Supplementary Table 6).

### Child protection/safeguarding procedures

Recommendations related to safeguarding or child protection procedures were included in almost half of the included documents (*n* = 45) (Table 4 and Supplementary Table 6). A key recommendation was that any agency with concerns about risk to an unborn or new-born baby should make a referral to children’s social care / social work where parental substance use was identified. Additionally, five documents outlined that if a child is born unexpectedly with NAS, an immediate referral to social work must be made [45, 47, 89, 94, 133]. Clear guidance around information sharing between agencies was often provided, emphasising that confidentiality was over-ridden in the interest of protecting the child. Some documents also stated women should be informed of what information was being shared and where possible, this should be done with their consent [71, 104, 108, 124, 134].

Of the 14 documents which contained recommendations for the timing of child protection case conferences and pre-birth assessments there was some variation. While some documents only stated that these should be conducted early on in maternity proceedings, [54, 63] most specified that these be conducted before or by 28 weeks [33, 52, 67, 94, 124] with some also noting that conferences should occur within 21 days of a late notification [64, 129] or with 28 calendar days of a concern being raised [82]. Other documents recommended that conferences be held between 28 and 32 weeks [71], or before 32 weeks gestation [107, 113, 127]. Clear multi-agency care plans were to be co-ordinated by a lead professional and where child protection concerns were identified it was recommended they were social work led, and that the plan be communicated clearly with the parents. It was also suggested a discharge planning meeting be held prior to a woman and baby leaving the hospital, with a full discharge care plan in place for all scenarios including when a baby needed to be accommodated separately from the mother (Table 4 and Supplementary Table 6).

Involving the father and wider family in any assessment was generally encouraged, and four documents stated the parenting skills of fathers should be assessed alongside mothers [43, 61, 71, 96].

### Specific recommended Interventions

There were only a few recommendations for use of psychosocial interventions with prescribed modes of delivery. These included: Parents under Pressure [44]; Intensive parenting programmes [64]; Care planning / Care co-ordination approach [46]; Safe & Together Intervention model (Domestic Abuse) [59, 137]; Community hub model [70]; Peer support intervention programme [91] and motivational interviewing [64, 131](Table 4 and Supplementary Table 6).

## Discussion

This scoping review sought to map UK clinical guidelines, treatment protocols and good practice guidance for optimising outcomes and reducing inequalities for women who use or are in treatment for drug use during the perinatal period. Overall, included guidance and policy documents made consistent suggestions regarding best practice; for example, multi-agency working, information sharing, and clear referral pathways. The importance of engaging women in antenatal services was frequently stressed, and they were often identified as a vulnerable or disadvantaged population (Table 3). There were references to adopting a non-judgemental, holistic approach that considered wider social, economic, and psychological issues (for example, housing, domestic abuse), and within more recent documents, a trauma-informed care approach was advocated. There were consistent recommendations relating to detoxification and intrapartum care.

There were a few notable differences between documents, which are worth highlighting, as variations in recommended practice such as these could result in inconsistencies in practice. For example, the NICE guidance [85] updated in 2022, states neither methadone or buprenorphine adversely affect neonatal outcomes, although they refer to emerging evidence that buprenorphine results in less severe NAS, which is consistent with the 2014 WHO Guidelines [135]. Although most OST prescribing guidelines recommended methadone, a recent document from NHS Highland [131] suggested buprenorphine was preferred as there have been associated poor physical health outcomes for babies born to mothers prescribed methadone. Recent systematic review evidence suggests buprenorphine has better health outcomes for infants than methadone [138]. This highlights the need for guideline developers to ensure recommendations are informed by the most up to date evidence and are reviewed regularly as research evidence can change quickly, and its application can be complex as it may be dependent upon the women’s individual circumstances. There was also lack of consensus identified around timescales and protocols for drug testing women in pregnancy, the lengths of time neonates should be observed for NAS and timings of case conferences and pre-birth assessments, further highlighting the need for available evidence to be reviewed and women’s experiences to be captured to support policy and guidelines.

More recent clinical guidelines emphasised adopting a holistic, trauma-informed care approach toward mothers. Involving partners and the wider family in the assessment and care planning process was also a key suggestion supported by the wider child protection, substance use, and domestic abuse policy context. For example, the Scottish Government family policies such as GIRFEC [139], The Best Start, [140] Rights, Respect and Recovery [102], and Women’s Health Plan [141] at a very broad level also advocated holistic assessment and trauma-informed care approaches. Many of the safeguarding documents identified from England which often drew on findings from the ‘Hidden Harm’ report [142] seemed to have a greater focus on risk of harm to the unborn and new-born baby. While some emphasise substance use does not necessarily mean parents are unable to provide ‘good enough’ parenting [29, 81], children’s social care policy documents in Scotland, and England and Wales nevertheless suggest women’s drug use in pregnancy can be a form of neglect [82, 132]. This focus on *harm* has been recognised in a recent policy review by Whittaker et al., [10] and could contribute to women’s reluctance to engage with services for fear of being stigmatised, and having their baby removed from their care [143, 144]. Balancing the complexity of the many medico-legal issues surrounding women who use or are in treatment for drug use and their babies such as the protection of the un-born child, and the mothers needs and rights raises many ethical issues. However, as Lupton (2012) has argued in regard to good practice and clinical guidelines, the mother’s needs and rights are often de-prioritised vis-a-vis those of the child [145].

There was a lack of guidance in relation to supporting women whose babies have been removed from their care suggesting a gap in policy for the support of these women who are recognised as being at high risk of suicide and drug overdose [146, 147]. Although guidelines currently in development outlines the need to support parents whose babies are removed from their care [148], they are not specific to women who use, or are in treatment for drug use or focused upon identifying or supporting the mental health needs of these women.

Despite the explicit recognition in many documents that domestic abuse may be a compounding factor for this population of women, there was a general lack of any specific recommendations for practitioners on how to involve fathers and yet remain vigilant about, and assess women’s exposure to, domestic abuse. There was one exception; ‘Families Affected by Drug and Alcohol Use in Scotland: A Framework for Holistic Whole Family Approaches and Family Inclusive Practice [59], the Scottish Government (2021) policy document which recommends using the Safe & Together model [137].

Within health and social care professions there is an expectation that policy and practice will be ‘evidence-based’ [149, 150]. The WHO guidance aimed at perinatal drug use [135], for example provided an evidence review for each of its included recommendations and categorises the strength of a recommendation based upon both the evidence and its applicability across different contexts. However, these guidelines are only cited in eight included documents [41, 54, 73, 77, 85, 97, 105, 117]. The UK NICE Guidelines ‘Pregnancy and complex social factors’ does not cite WHO (2014) guidance, but does include systematic review and RCT evidence, although it is unclear which specific recommendations they support. Only 28% of included documents cited systematic review or meta-analysis evidence, 53% referred to other guidelines, and the most frequently cited were NICE guidelines [34, 95, 151] (Supplementary Table 6).

The variability in how guideline documents are created has been recognised [149, 152] and is not particular to those included within this study. Although literature suggests guideline development should involve collaboration with representatives from those impacted by the subject [149], only 19% of included documents reported consulting stake holders, either with the public, service users, or people with lived experience (Supplementary Table 6) with only 13% mentioning that they could be used by pregnant women or their families (Supplementary Table 4).

### Strengths and Limitations

This is the first scoping review of UK clinical guidance and related policies that address the care needs of women who use or are in treatment for drug use during the perinatal period. It provides new knowledge by identifying and synthesising current recommended best practice, as well as identifying potential gaps, and inconsistencies between the documents. Additionally, and of relevance to the creators of guidelines and policy documents, it presents an overview of the evidence upon which the included documents are based, together with an insight into how they were created. The review was conducted by a team following rigorous process, and registered protocol [21].

As is the case with scoping reviews more generally, despite adopting a systematic approach to searching and screening, identifying documents to be included was an iterative process and there may be documents that were missed or published since the conclusion of the search. We acknowledge we only found one Welsh document which met the inclusion / exclusion criteria despite purposive searching and specific enquiries to experts in the field based within Wales. We also note that although our scoping of UK good practice and clinical guidelines is limited to the UK context our findings, particularly our observation that guidelines should be informed by the most up to date evidence, are likely to have relevance for other national settings. Furthermore, although seeking to identify documents within a set timeframe provides a reliable snapshot of the guidelines in use at the time of the search, and allowed us to answer or research question, it does not allow for the contextualisation of how these documents have changed over time. Key documents that were identified and published post conclusion of the review have been considered in our discussion.

It is possible that another limitation of the scoping review methodology is the omission of quality appraisal and the lack of prescriptive arguments [16]. However, what is presented here provides a robust overview of what clinical guidelines and policy documents suggest best practices to be in supporting women who use drugs during the perinatal period and will, as such, prove a valuable resource to both practitioners and policy decision makers [153]. Additionally, this review was undertaken as part of a much larger study (NIHR130619), and the authors are also conducting a mixed method systematic review of the intervention literature which will assist future guideline developers and policy makers in identifying the evidence base.

## Conclusions & recommendations

In this scoping review to map UK clinical and best practice guidelines for the care of women who use or are in treatment for drug use in the perinatal period we found consistent messages for professionals based on a core range of primary documents that were referred to. We identified and mapped broad range of best practice recommendations, providing a valuable resource for service providers and practitioners alike. However, we also identified gaps that highlight the need for the development of clinical and best practice guidelines in the UK that are (1) coproduced with women with experience of drug use in pregnancy (2) based on research evidence for approaches that improve outcomes for pregnant women who use or are in treatment for drug use; and (3) also address the support needs of postnatal women who have their baby removed from their care. This review also supports the need for and will inform our systematic review of the research literature to establish which treatment approaches and models of care there is evidence to support.

### Supplementary Information

**Additional file 1: Supplementary Table 1.** Database specific search strategy.

**Additional file 2: Supplementary Table 2.** Organisations contacted.

**Additional file 3: Supplementary Table 3.** Reasons for exclusion.

**Additional file 4: Supplementary Table 4.** Intended users.

**Additional file 5: Supplementary Table 5.** Relevance.

**Additional file 6: Supplementary Table 6.** Evidence.

## Data Availability

No additional data available.

## References

[1] European Monitoring Centre for Drugs and Drug Addiction (2014). Pregnancy and opioid use: strategies for treatment.

[2] Arpa S (2017). Women who use drugs: issues, needs, responses, challenges and implications for policy and practice.

[3] Public Health England. Adult substance misuse treatment statistics 2019 to 2020: report. UK Government; 2020. Available from: https://www.gov.uk/government/statistics/substance-misuse-treatment-for-adults-statistics-2019-to-2020/adult-substance-misuse-treatment-statistics-2019-to-2020-report. Accessed 05 Sept.

[4] Scobie G, Woodman K (2016). Evidence briefing on interventions to reduce illicit drug use during pregnancy (and in the postpartum period).

[5] Broadhurst K, Alrouh B, Mason C (2018). Born into care - newborns in care proceedings in England.

[6] Alrouh B, Broadhurst K, Cusworth L (2019). Born into care: newborns and infants in care proceedings in Wales.

[7] Hill L, Gilligan R (2020). How did kinship care emerge as a significant form of placement for children in care? A comparative study of the experience in Ireland and Scotland. Child Youth Serv Rev.

[8] Weber A, Miskle B, Lynch A (2021). Substance use in pregnancy: identifying stigma and improving care. Subs Abuse Rehabil.

[9] National Institute for Health and Care Excellence (2019). Intrapartum care for women with existing medical conditions or obstetric complications and their babies.

[10] Whittaker A, Martin F, Olsen A (2020). Governing parental drug use in the UK: what’s hidden in hidden harm?. Contemp Drug Probl.

[11] Faherty LJ, Kranz AM, Russell-Fritch J (2019). Association of punitive and reporting state policies related to substance use in pregnancy with rates of neonatal abstinence syndrome. JAMA Netw Open.

[12] Du Rose N (2015). The governance of female drug users: women’s experiences of drug policy.

[13] UK Government. Domestic Abuse Act Chapter. 17. Section 1. United Kingdom; 2021.

[14] Scottish Government. Domestic Abuse (Scotland) Act, ASP 5. Section 2. Scotland; 2018.

[15] Levac D, Colquhoun H, O’Brien K (2010). Scoping studies: advancing the methodology. Implement Sci.

[16] Arksey H, O’Malley L (2005). Scoping studies: towards a methodological framework. Int J Soc Res Methodol Theory Pract.

[17] Munn Z, Pollock D, Hanan K (2022). What are scoping reviews? Providing a formal definition of scoping reviews as a type of evidence synthesis. JBI Evid Synth.

[18] Peters M, Godfrey C, McInerney P et al. Chapter 11: scoping reviews. JBI Manual for evidence synthesis. 2020. 10.46658/JBIMES-20-12.

[19] Peters M, Godfrey C, Khalil H (2015). Guidance for conducting systematic scoping reviews. Int J Evid Based Healthc.

[20] Peters M, Godfrey C, Khalil H, et al. Chapter 11: scoping reviews. In: Aromataris E, Munn Z, editors. The Joanna Briggs Institute reviewer’s manual. Adelaide: The Joanna Briggs Institute; 2017.

[21] Radcliffe P, Gilmour L, Honeybul L et al. Scoping review protocol: mapping clinical guidelines, and policy documents that address the needs of women who are drug dependent during the perinatal period. 2021. 10.17605/OSF.IO/ZG8SM. 10.1186/s12884-023-06172-6 PMC10809451 38273236

[22] Tricco A, Lillie E, Zarin W (2018). PRISMA Extension for Scoping Reviews (PRISMA-ScR): checklist and explanation. Ann Intern Med.

[23] Smith H, Harvey C, Portela A (2022). Discharge preparation and readiness after birth: a scoping review of global policies, guidelines and literature. BMC Pregnancy Childbirth.

[24] Crowther S, MacIver E, Lau A (2019). Policy, evidence and practice for post-birth care plans: a scoping review. BMC Pregnancy Childbirth.

[25] Gilmour L, Maxwel M, Duncan E. Policy addressing suicidality in children and young people: an international scoping review. BMJ Open. 2019;9. 10.1136/bmjopen-2019-030699. 10.1136/bmjopen-2019-030699 PMC6830632 31662375

[26] NHS Greater Glasgow and Clyde. Use of alcohol and other drugs in pregnancy: guideline for management flowchart. NHS Greater Glasgow and Clyde; 2016. Available from: https://obsgynhandbook.nhsggc.org.uk/nhsggc-obstetrics-gynaecology-guidelines/guidelines-library/antenatal-general/cg-use-of-alcohol-and-other-drugs-in-pregnancy-guideline-for-management-flowchart/. Accessed 22 Mar.

[27] NHS Greater Glasgow and Clyde. Use of benzodiazepines in pregnancy: guideline for obstetric management. NHS Greater Glasgow and Clyde; 2016. Available from: https://obsgynhandbook.nhsggc.org.uk/nhsggc-obstetrics-gynaecology-guidelines/guidelines-library/antenatal-general/cg-use-of-benzodiazepines-in-pregnancy-guideline-for-obstetric-management/. Accessed 22 Mar.

[28] NHS Greater Glasgow and Clyde. Use of opiates in pregnancy: guideline for obstetric management. NHS Greater Glasgow and Clyde; 2016. Available from: https://obsgynhandbook.nhsggc.org.uk/nhsggc-obstetrics-gynaecology-guidelines/guidelines-library/antenatal-general/cg-use-of-opiates-in-pregnancy-guideline-for-obstetric-management/. Accessed 22 Mar.

[29] Hulme A, Galvani S (2019). A child’s first 1000 days: the impact of alcohol and other drugs. A BASW pocket guide for social workers.

[30] Centre for Early Child Development (2021). University of Birmingham, University of Buffalo. A good practice guide to support implementation of trauma-informed care in the perinatal period.

[31] Department of Health Social Services and Public Safety (2012). A strategy for maternity care in Northern Ireland 2012 ­- 2018.

[32] University Hospitals Birmingham NHS Foundation Trust. Abstinence syndrome. Clinical guideline. Birmingham: University Hospitals Birmingham NHS Foundation Trust; 2019.

[33] Mackie M, Brown R, Halliday P (2020). Additional support pathway for women with vulnerabilities.

[34] National Institute for Health and Care Excellence. Antenatal and postnatal mental health: clinical management and service guidance. England: National Institute for Health and Care Excellence; 2014 (reviewed 2020). Document No.: CG192.

[35] National Institute for Health and Care Excellence. Antenatal care. England: National Institute for Health and Care Excellence; 2021. Document No.: NG201.

[36] Royal College of Obstetricians and Gynaecologists (2012). Bacterial sepsis following pregnancy.

[37] Lingford-Hughes AR, Welch S, Peters L (2012). BAP updated guidelines: evidence-based guidelines for the pharmacological management of substance abuse, harmful use, addiction and comorbidity: recommendations from BAP. J Psychopharmacol.

[38] Bristol North Somerset and South Gloucestershire Clinical Commissioning Group. Benzodiazepines and Z-drugs as hypnotics and anxiolytics. Bristol: NHS Bristol North Somerset and South Gloucestershire; 2021.

[39] Public Health England (2017). Better care for people with co-occurring mental health and alcohol/drug use conditions.

[40] Care Quality Commission. Brief guide: substance misuse services – people in vulnerable circumstances. London: Care Quality Commission; 2018

[41] McAllister-Williams H, Baldwin D, Cantwell R (2017). British Association for Psychopharmacology Consensus guidance on the use of psychotropic medication preconception, in pregnancy and postpartum 2017. J Psychopharmacol.

[42] Naderian Z. Change, Grow, Live (CGL) procedure - substance misuse in pregnancy. Birmingham; 2019.

[43] Aberdeen A (2019). Drugs Partnership. Charter 3.2: births affected by drugs.

[44] National Institute for Health and Care Excellence (2017). Child abuse and neglect.

[45] Outer Hebrides Drug and Alcohol Partnership, Outer Hebrides Child Protection Committee (2018). Children affected by parental drug or alcohol related problems - GIRFEC oriented inter-agency guidelines.

[46] HIPS Safeguarding Children Partnership. 5.1 Children living in households where there is substance misuse. Hampshire, Isle of Wight, Portsmouth and Southampton Safeguarding Children’s Partnership; 2022. Available from: https://hipsprocedures.org.uk/hkyysz/parents-who-have-additional-needs/children-living-in-households-where-there-is-substance-misuse. Accessed 31 Aug 2022.

[47] Hull Safeguarding Children’s Partnership. Children of parents or carers that misuse substances. Hull Safeguarding Children’s Partnership; 2022. Available from: https://hullscb.proceduresonline.com/chapters/p_ch_par_misuse_subs.html. Accessed 31 Aug.

[48] Regional Child Protection Procedures for West Midlands. 2.3 Children of parents who misuse substances. Regional Child Protection Procedures for West Midlands; 2022 . Available from: https://westmidlands.procedures.org.uk/pkpzo/regional-safeguarding-guidance/children-of-parents-who-misuse-substances. Accessed 05 Sept.

[49] South Lankashire Partnership (2021). Children’s service plan: 2021–2023.

[50] HM Prison Service. Clinical services for substance users. London: HM Prison Service; 2000.

[51] National Institute for Health and Care Excellence (2016). Coexisting severe mental illness and substance misuse: community health and social care services.

[52] Derbyshire Safeguarding Children Board. Derby and Derbyshire multi-agency protocol for pre-birth assessments and interventions. Derbyshire; 2020.

[53] The Royal College of Psychiatrists, The Faculty of Forensic and Legal Medicine of the Royal College of Physicians (2020). Detainees with substance use disorders in police custody: guidelines for clinical management.

[54] Clinical Guidelines on Drug Misuse and Dependence Update 2017 Independent Expert Working Group (2017). Drug misuse and dependence: UK guidelines on clinical management.

[55] Peagram S. Drug misuse management in the acute hospital setting: guidelines. Doncaster and Bassetlaw: Doncaster and Bassetlaw Teaching Hospitals NHS Foundation Trust; 2017.

[56] National Collaborating Centre for Mental Health, National Institute for Health and Care Excellence. Drug misuse: opioid detoxification, the NICE guideline. London, Leicester: The British Psychological Society, The Royal College of Psychiatrists; 2019.

[57] National Health Service. Equity and equality: guidance for local maternity systems. United Kingdom: National Health Service; 2021.

[58] Lothian NHS (2021). City of Edinburgh Council, PrePare. Expressed breast milk - information for carers of vulnerable babies V1.0.

[59] Scottish, Government (2021). Minister for Drugs Policy. Families affected by drug and alcohol use in Scotland: a framework for holistic whole family approaches and family inclusive practice.

[60] North Lanarkshire CPC, South Lanarkshire CPC, Lanarkshire ADP (2015). Getting it right for children and families affected by parental alcohol and drug use in Lanarkshire.

[61] Whittaker A, Templeton L, Mitchell F (2013). Getting it right for children and families affected by parental problem alcohol and drug use: guidelines for agencies in Edinburgh and the lothians.

[62] Scottish Government, United Nations Convention on the Rights of the Child (2013). Getting our priorities right - updated good practice guidance for all agencies and practitioners working with children, young people and families affected by problematic alcohol and/or drug use.

[63] Forth Valley Alcohol & Drug Partnership, Falkirk Child Protection Committee, Clackmannanshire & Stirling Child Protection Committee. Getting our priorities right for children and families affected by parental alcohol and drug use: Guidance from the Forth Valley Alcohol and Drug Partnerships and Child Protection Committees. Forth Valley; 2019.

[64] East Ayrshire Child Protection Committee (2014). Good practice: working with pregnant women with parental substance misuse.

[65] Department of Health NI (2020). Guidance for Alcohol and Drug services in Northern Ireland to best deliver treatment and care during the COVID-19 pandemic. Northern Ireland.

[66] NHS Grampian (2019). Guidance for the use of buprenorphine products for the treatment of opioid dependence in NHS grampian.

[67] Barnsley Safeguarding Children Partnership. Guidelines for multi-agency assessment of pregnant women and their babies in cases where there is substance misuse. Barnsley: Barnsley Safeguarding Children Partnership; n.d. Available from: https://www.proceduresonline.com/barnsley/scb/p_preg_woment_sub_misuse.html. Accessed 31 Aug.

[68] Leeds City Council. 1.4.4 Guidelines for the assessment of parental substance misuse. Leeds: Leeds City Council; 2010 . Available from: https://leedschildcare.proceduresonline.com/chapters/p_assess_par_sub_mis.html. Accessed 31 Aug.

[69] St, Mungos. City of Westminster. Homeless pregnancy Toolkit. City of Westminster: St Mungos; 2017.

[70] NHS England. Implementing better births: a resource pack for local maternity systems. England: NHS England; 2017.

[71] Glasgow Child Protection Committee (2008). Inter-agency procedural guidance for vulnerable women during pregnancy.

[72] National Institute for Health and Care Excellence. Intrapartum care for healthy women and babies. England: National Institute for Health and Care Excellence; 2014. (reviewed 2017). Document No: CG 190.

[73] Wilson C, Boxall C, Demilew J (2019). Lambeth drug and alcohol service guidelines for the management of substance misuse in the perinatal period.

[74] Royal College of Obstetricians and Gynaecologists (2010). Late intrauterine fetal death and stillbirth.

[75] National Institute for Health and Care Excellence (2021). Looked-after children and young people.

[76] University Hospitals Birmingham NHS Foundation Trust. Management of substance misuse in pregnancy. Clinical guideline. Birmingham: University Hospitals Birmingham NHS Foundation Trust; 2020.

[77] Smith A, Lazenby C (2019). Management of substance use during pregnancy and the postnatal period.

[78] Royal College of Obstetricians and Gynaecologists (2011). Management of women with mental health issues during pregnancy and the postnatal period.

[79] Little M, Chapman M, Fairclough L (2019). Maternity services guideline: antenatal appointments guideline.

[80] Radomska M, Gibbings B, Bass J (2021). Maternity substance misuse in pregnancy guideline.

[81] Wolverhampton Safeguarding Children Board (2013). Hidden harm - parental substance misuse and the effects on children. Multi-agency guidelines.

[82] Scottish Government (2021). National guidance for child protection in Scotland 2021.

[83] The Highland Council, Highland NHS. North Highland vulnerable pregnancy pathway: taking a trauma informed approach in understanding and responding to vulnerability in pregnancy. Policy document. The Highlands: The Highland Council; 2020.

[84] The Bedside Clinical Guidelines Partnership, Staffordshire, Shropshire & Black Country Newborn and Maternity Network, Southern West Midlands Maternity and Newborn Network. Obstetric Guidelines 2017-2019. Advisory guidelines. England: Bedside Clinical Guidelines Partnership S, Shropshire & Black Country Newborn and Maternity Network and Southern West Midlands Maternity and Newborn Network; 2017.

[85] National Institute for Health and Care Excellence. Opioid dependence: scenario: managing special circumstances. Online: National Institute for Health and Care Excellence; 2022. Available from: https://cks.nice.org.uk/topics/opioid-dependence/management/managing-special-circumstances/. Accessed 05 Sept.

[86] Orkney Health and Care (2021). Orkney alcohol and drugs partnership strategy 2021-31.

[87] Outer Hebrides Community Planning Partnership (2020). Integrated children’s services plan 2020–2023. Policy document.

[88] Underdown A, Barlow J (2020). Parental emotional wellbeing and infant development.

[89] Milton Keynes Inter-Agency Safeguarding Children Procedures. Parental substance misuse. Milton Keynes Inter-Agency Safeguarding Children Procedures; 2022. Available from: https://mkscb.procedures.org.uk/ykyyyh/assessing-need-and-providing-help/parental-substance-misuse. Accessed 3 Mar.

[90] Public Health England. Parents with alcohol and drug problems: adult treatment and children and family services. [Web page]. UK Government; 2021. Available from: https://www.gov.uk/government/publications/parents-with-alcohol-and-drug-problems-support-resources/parents-with-alcohol-and-drug-problems-guidance-for-adult-treatment-and-children-and-family-services. Accessed 05 Sept.

[91] Scottish Government (2020). Perinatal & infant mental health programme board 2020–2021 delivery plan.

[92] Cooke J, Rutherford M (2018). Perinatal mental health: prescribing guidance for trust prescribers and GPs.

[93] National Institute for Health and Care Excellence (2021). Postnatal care.

[94] Dumfries and Galloway Strategic Pre-Birth Planning Group (2019). Pre-birth assessment protocol for vulnerable pregnancies.

[95] National Institute for Health and Care Excellence. Pregnancy and complex social factors: a model for service provision for pregnant women with complex social factors. England: National Institute for Health and Care Excellence; 2010. (reviewed 2018). Document No: CG 110. 31855334

[96] NHS Lothian (2011). Pregnancy and problem substance use.

[97] Kehoe M, Craven J, NHS Lothian Quality Prescribing Group (2016). Pregnancy guidance.

[98] Ministry of Justice, HM. Prison and Probation Service. Pregnancy, mother and baby units (MBUs), and maternal separation from children up to the age of two in women’s prisons. England: Ministry of Justice; 2021.

[99] Camden and Islington NHS Foundation Trust (2019). Prescribing guidance for substance misuse services.

[100] Department of Health Social Services and Public Safety (2020). Preventing harm, empowering recovery: a strategic framework to tackle the harm from substance use (2021-31).

[101] Royal College of Obstetricians & Gynaecologists (2015). Reducing the risk of venous thromboembolism during pregnancy and the puerperium.

[102] Scottish Government (2018). Rights, respect and recovery: Scotland’s strategy to improve health by preventing and reducing alcohol and drug use, harm and related deaths.

[103] Public Health England. Safeguarding and promoting the welfare of children affected by parental alcohol and drug use: a guide for local authorities online. UK Government; 2018. Available from: https://www.gov.uk/government/publications/safeguarding-children-affected-by-parental-alcohol-and-drug-use/safeguarding-and-promoting-the-welfare-of-children-affected-by-parental-alcohol-and-drug-use-a-guide-for-local-authorities. Accessed 21 Mar 2022.

[104] Rotherham Safeguarding Children Partnership. Safeguarding children of drug misusing parents: Rotherham Safeguarding Children Partnership. Available from: https://rotherhamscb.proceduresonline.com/chapters/p_chil_drug_mis_par.html. Accessed 05 Sept 2015.

[105] Cumbria Northumberland Tyne and Wear NHS Foundation Trust. Safeguarding children practice guidance note addiction services: pregnancy pathway and guidance. Cumbria Northumberland Tyne and Wear: Cumbria Northumberland Tyne and Wear NHS Foundation Trust; 2019.

[106] South Gloucestershire Safeguarding Children Board (2015). Safeguarding guidance for substance misuse.

[107] Rotherham Safeguarding Children Partnership. Safeguarding unborn and newborn babies. Rotherham Safeguarding Children Partnership; 2016. Available from: https://rotherhamscb.proceduresonline.com/chapters/p_sg_babies.html. Accessed 05 Sept.

[108] NHS England (2019). Saving babies’ lives care bundle: version two.

[109] Shetland Child Protection Committee (2017). Shetland’s integrated children’s services plan.

[110] Southwark Safeguarding Children Partnership (2020). Southwark joint service protocol to meet the needs of children and unborn children whose parents or carers have substance misuse problems.

[111] NHS Greater Glasgow & Clyde Alcohol and Drug Recovery Services Pharmacy Team (2019). Standards for drug & alcohol services in community pharmacies.

[112] Hall A, Parkes J (2019). Practice guidance: substance misuse in parents.

[113] Whittaker A, Lothian NHS (2003). Substance use in pregnancy. A resource pack for professionals in Lothian.

[114] Hull Safeguarding Children’s Partnership. Substance misuse in pregnancy. Hull Safeguarding Children’s Partnership; 2022. Available from: https://hullscb.proceduresonline.com/chapters/p_sub_mis_preg.html. Accessed 31 Aug.

[115] NHS University Hospitals of Leicester (2019). Substance misuse in pregnancy – guidance for the care of pregnant drug /alcohol users and their babies.

[116] Karkhanis P. Substance misuse in pregnancy. Birmingham: Heart of England NHS Foundation Trust; 2016

[117] Mulay S (2017). Substance misuse in pregnancy and subsequent care of the newborn.

[118] Royal Cornwall Hospitals NHS Trust (2020). Substance misuse in pregnancy, labour and post delivery clinical guideline.

[119] Thompson C, Sharman N (2021). Substance misuse in pregnancy: multidisciplinary guidelines for Frimley Health NHS Foundation Trust.

[120] Welsh Government (2011). Substance Misuse Treatment Framework (SMTF): guidance for evidence based community prescribing in the treatment of substance misuse.

[121] Birmingham Women and Children Hospital Trust, Gray H. Substance misuse: management of pregnant women. Birmingham: Birmingham Women and Children Hospital Trust; 2021.

[122] Public Health England (2013). Supporting information for developing local joint protocols between drug and alcohol partnerships and children and family services.

[123] Angus Child Protection Committee (2021). Dundee City Child Protection Committee, Perth, Kinross Child Protection Committee. Tayside multi-agency practitioner’s guidance: concern for unborn babies.

[124] Robertson C (2019). Multi-agency guidance for staff: the management of high risk pregnancies.

[125] National Collaborating Centre for Mental Health (2018). The perinatal mental health care pathway; full implementation guidance.

[126] Folliard K (2021). Care of vulnerable women in pregnancy.

[127] Hull Safeguarding Children’s Partnership. Unborn procedures and guidance (Pre-birth pathway): Hull Safeguarding Children’s Partnership,; 2022. Available from: https://hullscb.proceduresonline.com/chapters/p_unborn.html. Accessed 31 Aug.

[128] Scottish Government (2015). Universal health visiting pathway in Scotland: pre-birth to pre-school.

[129] Bell M, Burns D (2017). Vulnerable pregnancy procedure.

[130] West Yorkshire Consortium Inter Agency Safeguarding and Child Protection Procedures. 1.4.15 Children of Drug Misusing Parents Online: West Yorkshire Consortium; 2022 . Available from: https://westyorkscb.proceduresonline.com/p_chil_drug_mis_par.html. Accessed May 2022.

[131] Harrington S, MacPhee C. Women, pregnancy and substance use: good practice guideline. The Highlands: NHS Highland, The Highland Council; 2019.

[132] UK Government (2018). Working together to safeguard children: a guide to inter-agency working to safeguard and promote the welfare of children. Department for Education.

[133] Derby and Derbyshire Safeguarding Children’s Partnership. Working with parents who are misusing substances: Derby and Derbyshire Safeguarding Children’s Partnership; 2022. Available from: https://derbyshirescbs.proceduresonline.com/p_wking_par_misusing_subs.html. Accessed: 31 Aug.

[134] Aberdeen City CPC Operational Subcommittee. Working with vulnerable unborn babies and their families - multi-agency practice guidance. Aberdeen: Aberdeen City Child Protection Committee; 2017.

[135] World Health Organization (2014). Guidelines for identification and management of substance use and substance use disorders in pregnancy.

[136] Keegan J, Parva M, Finnegan M (2010). Addiction in pregnancy. J Addict Dis.

[137] Mandel D (2013). Safe and together.

[138] Kinsella M, Halliday LOE, Shaw M (2022). Buprenorphine compared with methadone in pregnancy: a systematic review and meta-analysis. Subst Use Misuse.

[139] Scottish Government, Cabinet Secretary for Education and Skills, Minister for Children and Young People. Getting it right for every child (GIRFEC). Edinburgh: Scottish Government; 2019. Available from: https://www.gov.scot/policies/girfec/. Accessed 15 Aug.

[140] Scottish Government (2017). The best start: maternity and neonatal care plan executive summary.

[141] Scottish Government, Minister for Public Health, Women's Health and Sport. Women’s health plan - a plan for 2021–2024. Edinburgh: Scottish Government; 2021.

[142] UK Advisory Council on the Misuse of Drugs. Hidden harm: responding to the needs of children of problem drug users. London: UK Advisory Council on the Misuse of Drugs; 2003.

[143] Leiner C, Cody T, Mullins N (2021). The elephant in the room; a qualitative study of perinatal fears in opioid use disorder treatment in Southern Appalachia. BMC Pregnancy Childbirth.

[144] Stone R (2015). Pregnant women and substance use: fear, stigma, and barriers to care. Health Justice.

[145] Lupton D (2012). Precious cargo’: foetal subjects, risk and reproductive citizenship. Crit Public Health.

[146] Knight M, Bunch K, Tuffnell D (2021). On behalf of MBRRACE-UK. Saving lives, improving mothers’ care - lessons learned to inform maternity care from the UK and Ireland confidential enquiries into maternal deaths and morbidity 2017-19.

[147] Knight M, Bunch K, Patel R (2022). On behalf of MBRRACE-UK. Saving lives, improving mothers’ care core report - lessons learned to inform maternity care from the UK and Ireland confidential enquiries into maternal deaths and morbidity 2018-20.

[148] Mason C, Broadhurst K, Ward H (2022). Born into care: draft best practice guidelines when the state intervenes at birth (for feasibility testing) - Nuffield Family Justice Observatory.

[149] Andrews EJ, Redmond HP (2004). A review of clinical guidelines. Br J Surg.

[150] Shekelle PG, Woolf SH, Eccles M (1999). Developing guidelines. West J Med.

[151] National Institute for Health and Care Excellence. Alcohol-use disorders: diagnosis, assessment and management of harmful drinking (high-risk drinking) and alcohol dependence. London: National Institute for Health and Care Excellence; 2011. Contract No.: CG 115. 39808037

[152] Kredo T, Bernhardsson S, Machingaidze S (2016). Guide to clinical practice guidelines: the current state of play. Int J Qual Health Care.

[153] Peters MDJ, Marnie C, Colquhoun H (2021). Scoping reviews: reinforcing and advancing the methodology and application. Syst Rev.

