# Supplementary material for: Scoping review: mapping clinical guidelines and policy documents that address the needs of women who are dependent on drugs during the perinatal period
Source: BMC Pregnancy Childbirth. 2024 Jan 25;24:84. doi: 10.1186/s12884-023-06172-6 (PMC10809451; doi:10.1186/s12884-023-06172-6)
Supplement: Supplementary file 2 — Additional file 2: Supplementary Table 2. Organisations contacted. [file 12884_2023_6172_MOESM2_ESM.docx]

| **Expert** | **Organisation** | **Role** | **Country** |
| --- | --- | --- | --- |
| EACG member | Academic | Research Director – Addictions | Scotland |
| EACG member | Academic | Professor of Child Protection | England |
| EACG member | NHS | Child Health Commissioner | Scotland |
| EACG member | NHS | Maternity & Gynaecology Matron | England |
| EACG member | Voluntary Sector | Service Provider | England |
| EACG member | NHS | Specialist Midwife | England |
| EACG member | Institute of Health Visiting | Regional Lead | England |
| EACG member | Primary Health | GP | Scotland |
| EACG member | NHS | Service Provider | England |
| EACG member | Local Government | Public Health Specialist | England |
| EACG member | Voluntary Sector | CEO | England |
| EACG member | Public Health England | Parents, carers & families (alcohol & drugs)  Addictions & Inclusion | England |
| External expert | Academic | Director of Research | Northern Ireland |
| External expert | Public Health Agency | Midwifery Officer | Northern Ireland |
| External expert | Academic | Professor of Social Work | Wales |
| External expert | Academic | Reader of Social Sciences | Wales |
| External expert | Welsh Government | Substance Misuse Department | Wales |
| External expert | Neonatal Operational Delivery Network | Regional Lead | England |
| External expert | Academic | Lecturer | England |
| External expert | Academic | Professor of Psychiatry | England |
| External expert | Academic | Trainee Psychiatrist | England |
| External expert | Academic | Professor of Sociology | England |
| External expert | NHS | Addiction Psychiatrist | England |
| External expert | NHS | Addictions Psychologist | England |
| External expert | Voluntary Agency | CEO | England |
| External expert | NHS | Specialist Midwife | England |
| External expert | NHS | Lead Midwife (Safeguarding) | Scotland |
| External expert | Voluntary Agency | Policy and Practice Lead (Harm Reduction) | Scotland |
| External expert | NHS | Lead Midwife | Scotland |
| External expert | Voluntary Agency | Service Provider for women who are using substances in pregnancy and 6 months post birth | Scotland |
| External expert | Local Government | City Council Service for women who are pregnant and using drugs | Scotland |
| External expert | NHS | Alcohol and Drugs Partnership | Scotland |
| External expert | Voluntary Agency | Service Provider | Scotland |
| External expert | Scottish Government | Drugs Policy Division | Scotland |
| External expert | Local Government | Public Protection | Scotland |
| External expert | Local Government | Child Protection | Scotland |
| DMEC expert | Academic | Professor – School of Medicine | Scotland |
| DMEC expert | Academic | Senior Lecturer in Midwifery | England |
| DMEC expert | Academic | Professor of Addiction Biology | England |
| DMEC expert | Academic | Professor of Sociology | England |
| External expert | Academic | Professor of Harm Reduction | International |
